# Calycosin alleviates myocardial injury in diabetic mice by enhancing lipophagy in cardiomyocytes via Rab7a regulation

**DOI:** 10.3389/fphar.2026.1880993

**Published:** 2026-10-22

**Authors:** Qi Zhang, Mingyu Li, Xiaowan Zhao, Yuchun Wang, Yiwen Jiang, Yu Feng, Ming Wang, Honglian Zhang, Pengling Ge, Jianwen Zhou, Jicheng Liu

**Affiliations:** 1 College of Pharmacy, Qiqihar Medical University, Qiqihar, Heilongjiang, China; 2 Heilongjiang University of Chinese Medicine, Harbin, Heilongjiang, China

**Keywords:** calycosin, diabetic cardiomyopathy, lipid droplets, lipophagy, ras-related protein Rab-7a

## Abstract

**Background:**

Diabetic cardiomyopathy (DCM) is a major life-threatening cardiovascular complication of diabetes mellitus, characterized by myocardial lipid deposition, lipotoxicity and progressive cardiac dysfunction. Impaired lipophagy—the selective autophagic clearance of lipid droplets (LDs)—is a key driver of DCM progression, and Rab7a, a small GTPase, is essential for autophagosome–lysosome fusion, the rate-limiting step of efficient lipophagic degradation. Calycosin (CA), a natural isoflavone derived from *Astragalus membranaceus*, exerts antidiabetic and cardioprotective effects. However, whether CA regulates lipophagy via Ras-related protein Rab-7a (Rab7a) to ameliorate DCM remains largely unknown. The present study investigated whether CA alleviates DCM by promoting lipophagy through Rab7a-dependent mechanisms.

**Methods:**

*In vivo*, DCM was induced in male C57BL/6J mice by a high-fat diet combined with low-dose streptozotocin (STZ), followed by daily CA administration. *In vitro*, neonatal mouse cardiomyocytes (NMCMs) were isolated and exposed to high glucose and high lipid (HGHL) conditions to mimic the DCM microenvironment. Cardiac function was assessed by echocardiography. Myocardial lipid accumulation was evaluated by Oi-Red O staining, Bodipy staining and triglyceride (TG) quantification assay. Lipophagy was monitored using transmission electron microscopy (TEM), LC3-LD colocalization analysis and Western blotting. Rab7a was knocked down in NMCMs using specific small interfering RNAs (siRNAs), and its ubiquitination level was detected by co-immunoprecipitation (Co-IP) assay.

**Results:**

CA significantly improved cardiac function, reduced myocardial fibrosis and lipid accumulation, and restored impaired lipophagic flux in DCM mice. Consistently, in HGHL-stimulated NMCMs, CA promoted lipophagy and suppressed lipotoxic injury. Mechanistically, CA upregulated Rab7a protein expression and inhibited its ubiquitination. Rab7a knockdown abolished CA-mediated lipophagy activation and subsequent lipid clearance.

**Conclusion:**

CA enhances cardiomyocyte lipophagy by upregulating Rab7a and suppressing its ubiquitination, thereby mitigating excessive lipid deposition and subsequent myocardial injury in DCM mice. These findings reveal a novel mechanism by which CA regulates cardiomyocyte lipophagy and highlight Rab7a-mediated lipophagy as a promising therapeutic target for diabetic cardiac lipotoxicity.

## 1 Introduction

Diabetic cardiomyopathy (DCM) is a common complication of diabetes and represents a cause of mortality among diabetic patients (Yang M. et al., 2018). Lipid abnormalities affect 60%–70% of individuals with type 2 diabetes mellitus, leading to ectopic lipid deposition and cardiovascular damage (Tang et al., 2022). Pathological lipid accumulation in cardiomyocytes induces lipotoxicity, which exacerbates myocardial injury, dysfunction, and ultimately heart failure (Christian, 2009; Zhang et al., 2015; Wen et al., 2019; Lopaschuk et al., 2010). Therefore, the clearance of lipid droplets (LDs) is essential for the preservation of cardiac function.

Studies have demonstrated the critical role of lipophagy in eliminating surplus LDs (Ke et al., 2024; Chen et al., 2025; Li et al., 2026; Berardi et al., 2022). Lipophagy, a specialized form of autophagy, selectively targets LDs and is essential for intracellular lipid metabolism (Guan et al., 2022; Michal et al., 2024), impacting cellular energy balance and maintaining intracellular lipid concentrations. Studies have demonstrated that during lipid overload in DCM, lipophagy activation promotes the breakdown of excess LDs, safeguarding cardiomyocytes against lipotoxic damage (Tabibzadeh, 2023). Nevertheless, autophagic activity is decreased in DCM (Xu et al., 2013; Kanamori et al., 2015), underscoring the significance of restoring and upregulating lipophagy. Among the regulatory molecules involved, Rab7a plays a crucial role in the membrane fusion process during autophagy (Singh and Cuervo, 2011). By interacting with lysosomal membrane proteins, such as LAMP1, and SNARE complexes, including VAMP7 and Syntaxin 7, Rab7a facilitates the fusion of autophagosomes with lysosomes, resulting in the formation of autolysosomes (Qiang et al., 2022). This process is vital for the degradation of LDs, thereby offering new avenues for the development of preventive pharmaceuticals.

Calycosin (CA), a bioactive compound extracted from the roots of *Astragalus membranaceus*, exhibits multiple pharmacological properties (Liu et al., 2022; Yosri et al., 2022). Research suggests that CA affects cardiovascular conditions by inhibiting foam cell formation through the enhancing autophagy (Hu et al., 2023; Wu et al., 2024). However, further investigations are required to determine whether CA can alleviate cardiomyocyte injury by regulating lipophagy.

The present study demonstrated that CA exerts cardioprotective effects against myocardial injury in DCM mice by enhancing lipophagy, using a combination of *in vivo* and *in vitro* experiments. The present study further explored the potential molecular interaction between CA and Rab7a, and unraveled the underlying mechanism by which CA ameliorates DCM. These findings provide a novel and promising therapeutic strategy for DCM treatment.

## 2 Methods

### 2.1 Animals experiment

The experiment used 8-week-old male C57BL/6J mice (Changsheng Biological Co., Ltd., Liaoning, China). The animals were housed in standard laboratory conditions (22 °C–24 °C light/dark cycle for 12 h) and fed standard rodent feed and water. All animal experiments are conducted under legal practice guidelines and regulations.

Mice were anesthetized by intraperitoneal injection of 1.25% Avertin (tribromoethanol, 250 mg/kg body weight) followed by cervical dislocation. Death was verified by the absence of respiratory movement and lack of response to paw pinch. The animal study protocol was approved by the Institutional Review Board of Qiqihar Medical University and conducted in accordance with the guidelines of the Institutional Animal Care and Use Committee of Qiqihar Medical University (approval number: QMU-AECC-2025–103).

### 2.2 DCM mouse model establishment

A total of 40 male C57BL/6J mice (8 weeks old) were initially allocated, with 10 mice per group (CTL, CTL + CA, DCM, and DCM + CA). Mice in the CTL and CTL + CA groups were fed a standard diet, whereas mice in the DCM and DCM + CA groups were fed a high-fat diet (HFD) for 4 weeks to induce insulin resistance. Subsequently, T2DM was induced in the DCM and DCM + CA groups by intraperitoneal injection of streptozotocin (STZ; Sigma-Aldrich, United States) at 30 mg/kg for five consecutive days. The CTL and CTL + CA groups received equivalent volumes of sodium citrate buffer (SCB; Solarbio, Beijing, China), the solvent for STZ. One week after the final STZ injection, fasting blood glucose levels were measured via tail vein sampling; mice with blood glucose concentrations exceeding 16.7 mmol/L were confirmed to have successfully developed T2DM. Mice with blood glucose <16.7 mmol/L or that died during the induction period were excluded. Thereafter, mice in the CTL + CA and DCM + CA groups were orally administered CA (Shanghai Yuanye Bio-Technology Co., Ltd., Shanghai, China) at 10 mg/kg daily for 4 weeks. CA was dissolved in 0.9% normal saline. The CTL and DCM groups received an equivalent volume of 0.9% normal saline as the vehicle control. The final sample sizes were n = 8 per group for echocardiography and n = 4 per group for biochemical and histological analyses.

### 2.3 Echocardiographic

12 weeks after diabetes induction, cardiac function was assessed using a VINNO D650 LAB high-resolution small-animal ultrasound imaging system (VINNO Technology Co., Ltd., Jiangsu, China). Mice were anesthetized with 1% isoflurane, and heart rate was continuously monitored during image acquisition. Two-dimensional (2D), M-mode, and pulsed-wave (PW) Doppler echocardiography were performed. Standard parasternal long-axis and short-axis views at the papillary muscle level were obtained. LVIDd and LVIDs were measured from M-mode images, and FS and EF were automatically calculated by the built-in software using the Teichholz algorithm. For diastolic function assessment, PW Doppler recordings were obtained from the apical four-chamber view with the sample volume positioned at the tips of the mitral valve leaflets to measure peak early (E) and late (A) transmitral inflow velocities, and the E/A ratio was calculated. All measurements were performed by a single investigator blinded to the experimental groups. Three consecutive cardiac cycles were analyzed for each mouse, and the average value was used for statistical analysis. Mice exhibiting echocardiographic evidence of left ventricular structural and functional abnormalities were considered to have developed DCM.

### 2.4 LDH assay

Serum and cell supernatants were collected following centrifugation. For the assay, double-distilled water, sodium pyruvate standard solution, test sample, and Reagents 1–4 were sequentially added to the blank, standard, experimental, and control wells, respectively. After incubation at 37 °C, sodium hydroxide solution was added and the mixture was vortexed sufficiently. The reaction was allowed to stand at room temperature for a specified duration before measuring the absorbance at 440 nm using a microplate reader. Calculations were performed in according with the manufacturer’s instructions (Nanjing Jiancheng Bioengineering Institute, Nanjing, China) and standard protocols.

### 2.5 Histological analysis and Oil-Red O staining

After echocardiographic analysis, tissues were collected. Portions of the tissue samples were fixed in 4% paraformaldehyde, dehydrated, and embedded in paraffin for hematoxylin-eosin (HE) (Liaoning Baihao Biotechnology, China) and Masson’s trichrome staining (Solarbio, Beijing, China). Another portion was snap-frozen in liquid nitrogen for cryosectioning and subsequent Oil-Red O staining (Beyotime Biotechnology, Shanghai, China).

### 2.6 Triglyceride content assay

Serum samples and homogenized myocardial tissue supernatants were collected, and the corresponding extraction reagents were added to each tube according to the manufacturer’s instructions. The mixtures were thoroughly mixed and vortexed to allow phase separation. The upper organic phase was transferred to a new Eppendorf tube, and subsequent reaction reagents were added as specified by the manufacturer (Solarbio, Beijing, China). After incubation in a water bath and cooling to room temperature, the absorbance was measured at 420 nm, and triglyceride (TG) concentrations were calculated accordingly.

### 2.7 Transmission electron microscopy

Isolated hearts were fixed in 3% phosphate-glutaraldehyde. Subsequently, the hearts were dehydrated through a graded series of ethanol solutions and finally embedded. After preparing ultra-thin sections, double staining with 3% uranyl acetate and lead citrate was performed, followed by observation and analysis under a transmission electron microscope (TEM).

### 2.8 Western blot assay

Neonatal mouse cardiomyocytes (NMCMs) and heart tissue were lysed in RIPA buffer (Beyotime Biotechnology, China) to extract total protein. Protein concentrations were determined using a BCA Protein Assay Kit (Beyotime Biotechnology, China). Protein samples (40 μg) were separated by SDS-PAGE, then transferred to a nitrocellulose membrane (Millipore, United States) and blocked with 5% non-fat milk. The membrane was incubated with primary antibodies at 4 °C overnight, followed by incubation with secondary antibodies (anti-rabbit IgG-HRP, Beyotime Biotechnology, China) at room temperature for 1.5 h in the dark. Immunoblots were detected and quantified using Image Studio software. Primary antibodies used were as follows: anti-LC3 antibody (1:1000) (Cell Signaling Technology, United States), anti-P62 antibody (1:1000) (Cell Signaling Technology, United States), anti-Rab7a antibody (1:1000) (Proteintech, Wuhan, China), and anti-Ubiquitin antibody (1:1000) (Proteintech, Wuhan, China).

### 2.9 Isolation and culture of NMCMs

NMCMs were isolated from 1- to 3-day-old C57BL/6J pups. Briefly, the hearts were surgically excised and immediately subjected to digestion with Trypsin-EDTA (Beyotime Biotechnology, China). Following neutralisation with DMEM supplemented with 10% fetal bovine serum, the cell suspensions were centrifuged at 1500 rpm for 5 min and then resuspended in DMEM. The cells were filtered through a 70 μm mesh filter, seeded into culture dishes, and incubated at 37 °C in a humidified incubator containing 5% CO_2_ for 2 h. NMCMs were subsequently isolated using the differential adhesion method and seeded into new culture dishes.

### 2.10 Cell model construction and drug formulation

NMCMs were isolated and cultured in low-glucose DMEM supplemented with 10% FBS and 1% penicillin-streptomycin at 37 °C in a 5% CO_2_ humidified incubator. Cells were randomly divided into four groups: the normal glucose (NG) group (5.5 mmol/L glucose); the high glucose and high lipid (HGHL) group treated with 25 mmol/L glucose and 150 μmol/L palmitic acid (PA); and two CA intervention groups treated with 5 μmol/L of CA under both NG and HGHL conditions. To prepare the PA treatment, a 50 mmol/L stock solution was dissolved in anhydrous ethanol, complexed with 10% BSA, and subsequently diluted to the working concentration using high-glucose DMEM.

### 2.11 MTT assay

Following 24 h of model establishment, 10 µL of MTT solution (5 mg/mL in PBS) was added to each well. The incubation proceeded for an additional 4 h, after which it was halted. The supernatant was discarded, and 150 µL of DMSO was added to each well. The plate was shaken to ensure complete mixing, conducted in the dark to prevent degradation of formazan. Absorbance was subsequently measured at 450 nm.

### 2.12 Bodipy lipid staining

NMCMs were seeded into a 24-well plate and allowed to adhere completely. Following full attachment, a cell injury model induced by HGHL was established, and subsequent experiments were performed after 24 h. Samples were processed in accordance with the manufacturer’s instructions (Beyotime Biotechnology, China). Bodipy 493/503 (Beyotime, C0034S) at 1:1000 dilution in PBS, incubated for 30 min at room temperature in the dark. Imaging was performed on an Olympus FV3000 confocal microscope with excitation at 488 nm and emission at 503–520 nm. Cellular images were observed and captured using a laser confocal microscope. Fluorescence intensity was quantified using ImageJ.

### 2.13 Colocalization of LC3 with LDs

NMCMs were plated into a 24-well plate, allowed to fully adhere, and then treated with HGHL and CA; after 24 h, cells were fixed with 4% paraformaldehyde for 15 min, washed three times with PBS, permeabilized with permeabilization buffer for 30 min followed by three PBS washes, blocked with blocking buffer at 37 °C for 60 min, and washed again with PBS. For colocalization: LC3 was detected with anti-LC3B antibody (CST, 3868S, 1:200), followed by Cy3-conjugated secondary antibody (Beyotime, A0516, 1:200; excitation 561 nm/emission 570–620 nm). LDs were stained with Bodipy 493/503 as above. Colocalization was quantified using Pearson correlation coefficient.

### 2.14 Molecular docking

The three-dimensional (3D) structure of CA was retrieved from the PubChem database and converted to PDB format using ChemDraw. The 3D structure of the target protein was obtained from the AlphaFold Protein Structure Database. Molecular docking and binding energy calculations were performed using AutoDock Vina. Finally, the docking results and binding interactions were visualized using PyMOL software.

### 2.15 Coimmunoprecipitation

NMVCs were treated with 5 μmol/L proteasome inhibitor (MG132) (Proteintech, Wuhan, China) for 6 h. Thereafter afterwards, cells were lysed for a coimmunoprecipitation (CO-IP) experiment carried out with Protein A/G Magnetic Beads (Med Chem Express, China) following the manufacturer’s instructions. Western blotting was performed to detect the ubiquitination level of Rab7a with anti-Ubiquitin antibody.

### 2.16 Transfection

NMCMs were seeded in culture dishes and cultured for 48 h. When cell confluency reached approximately 60%–80%, transfection was performed. Transfection complexes were prepared according to the manufacturer’s instructions (GENCEFE, China) The specific primer sequences utilized in the siRNA analysis can be found in Table 1.

**TABLE 1:** siRNA-specific primer sequence.

Gene name	Sequence (5′–3′)
Rab7a	F: GAGCGGACUUUCUGACCAATT R: UUGGUCAGAAAGUCCGCUCTT

### 2.17 Statistical analysis

All data were processed by GraphPad Prism 10.1.2 software (GraphPad Prism; San Diego, CA, United States), and all experiments were repeated at least three times. The results were presented as average value ±,standard error of the mean (SEM). Normality was assessed via Shapiro-Wilk test (all datasets n ≤ 50). For normally distributed data with homogeneous variances Levene’s test *p* ≥ 0.05), ordinary one-way ANOVA followed by Tukey’s post-hoc test was applied; if variances were unequal (*p* < 0.05), Welch’s ANOVA with Games-Howell post-hoc test was used; non-normal data were analyzed using Kruskal–Wallis test with Dunn-Bonferroni correction. Adjusted *p* < 0.05 were considered significant.

## 3 Results

### 3.1 CA reduced body weight and blood lipid in DCM mice

To establish the DCM model, mice were initially fed an HFD for 4 weeks to induce significant weight gain. After 4 weeks of HFD feeding, STZ was administered to induce pancreatic β-cell injury, and the mice were maintained on the same HFD until week 12 to allow development of the DCM model (Figure 1A). Following STZ injection, mice exhibited elevated blood glucose levels at all assessed time points. CA treatment significantly attenuated body weight gain in DCM model mice by week 10 (Figure 1B), accompanied by an approximately 31.6% reduction in serum TG levels (Figure 1C). However, no significant change in blood glucose levels was observed between the CA-treated group and the model group (Figure 1D).

**FIGURE 1:**
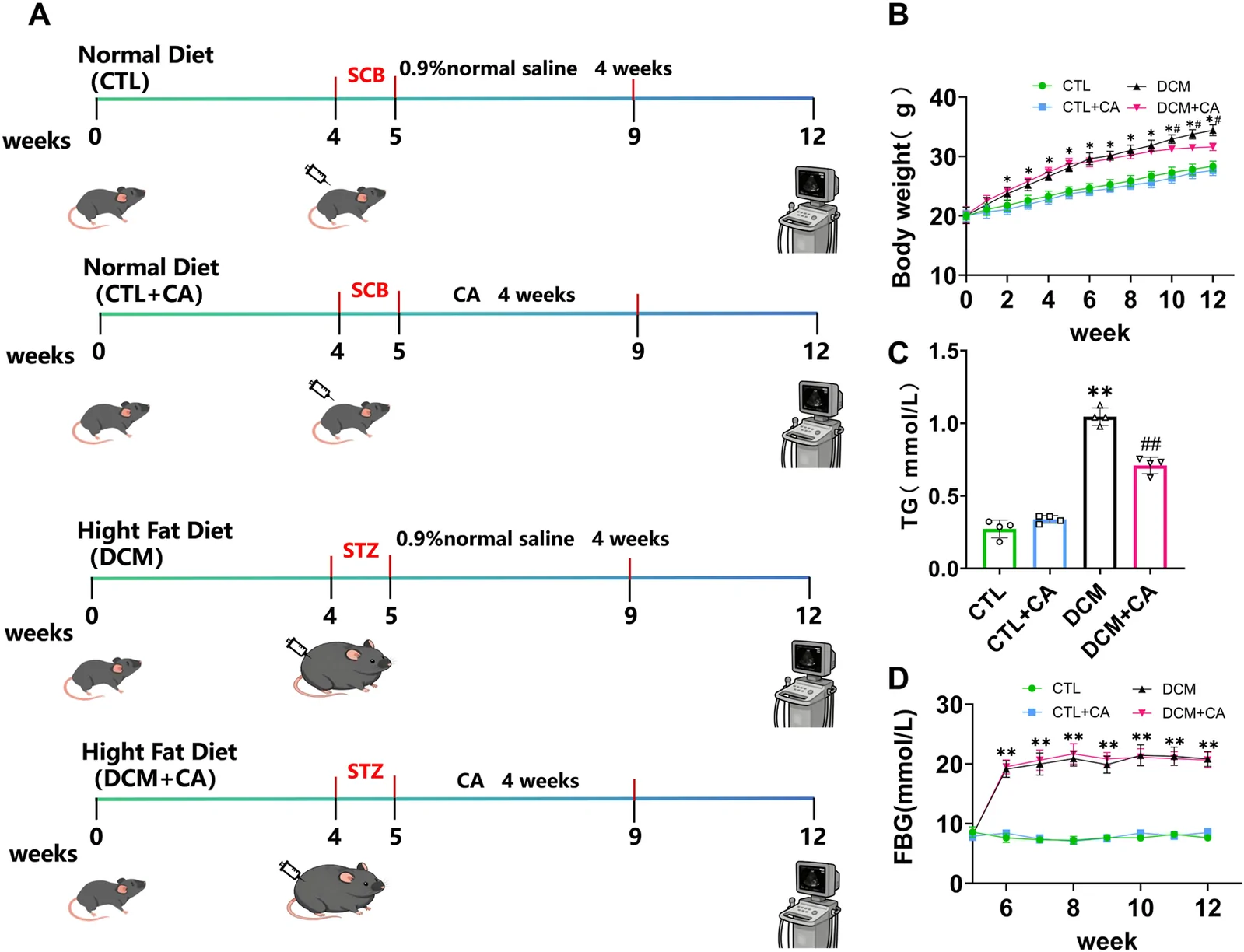
Effects of CA on body weight, serum TG and FBG in DCM mice. **(A)** Schematic diagram of the animal experimental protocol; **(B)** Body weight; **(C)** TG levels in mouse serum samples; **(D)** Fasting blood glucose. For body weight and FBG: n = 8; for serum TG: n = 4. *P < 0.05, **P < 0.01 vs. CTL, #*P* < 0.05, ##*P* < 0.01 vs. DCM. Data are presented as the mean ± SEM.

### 3.2 CA improved cardiac function and ameliorates cardiac remodeling in DCM mice

To investigate the cardioprotective role of CA in DCM, echocardiographic and histological analyses were performed. Following HFD/STZ induction, DCM mice exhibited a more pronounced reduction in the E/A ratio compared with the CTL group, indicating impaired diastolic function. There was a significant increase in LVIDd and LVIDs, alongside a significant reduction in EF and FS compared with the CTL group, indicating systolic dysfunction (Figures 2A,B). Elevated serum LDH levels further confirmed myocardial injury in DCM mice (Figure 2D). Additionally, histological analysis revealed adverse cardiac remodeling in DCM mice, characterized by disordered myocardial architecture, enlarged cardiomyocyte cross-sectional areas (Figure 2C), and increased myocardial interstitial collagen deposition. CA treatment significantly ameliorated these pathological changes, with an approximately 44.3% reduction in total collagen deposition (Figure 2E).

**FIGURE 2:**
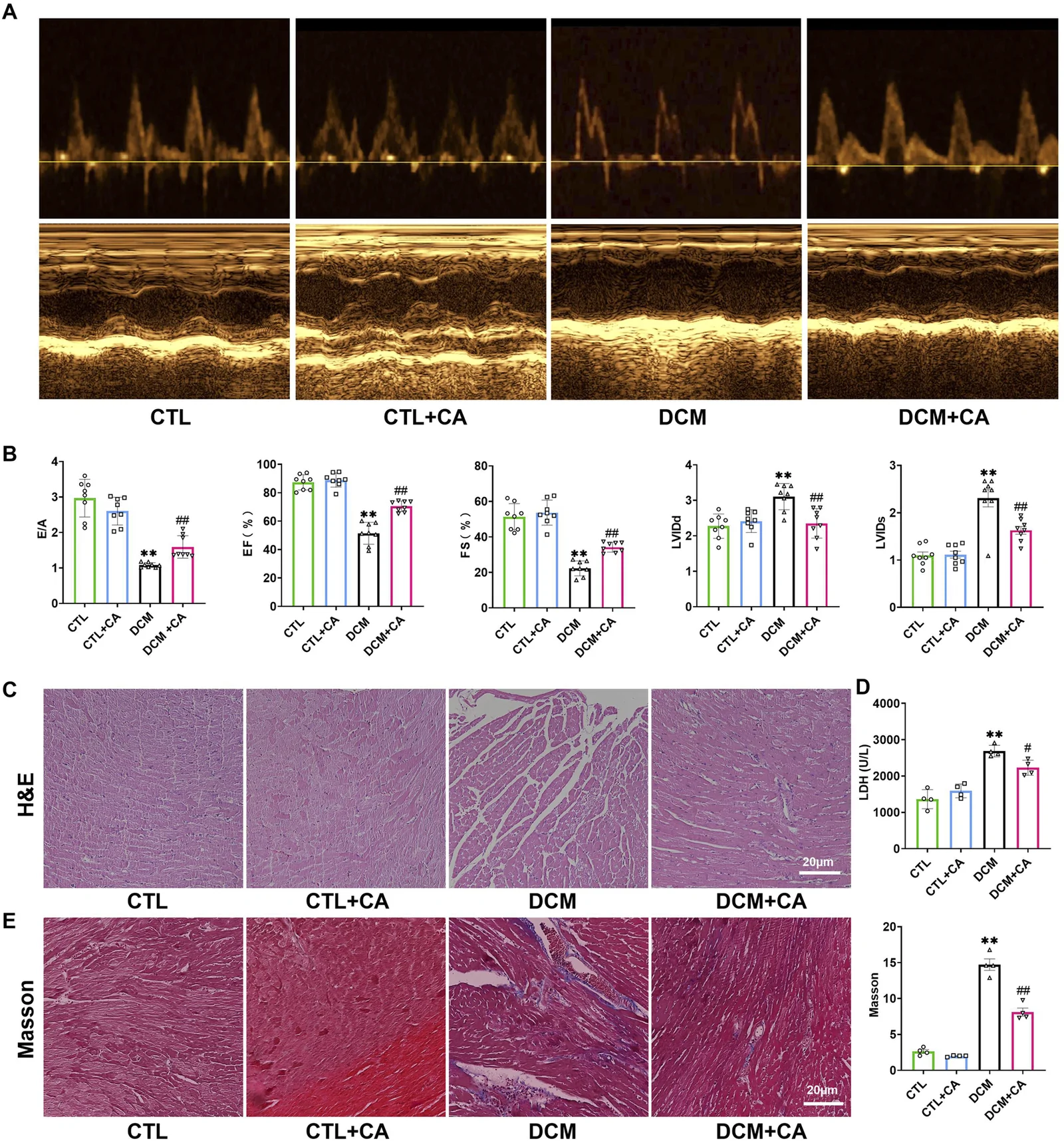
Effects of CA on cardiac function and adverse cardiac remodeling in DCM mice. **(A)** Representative echocardiographic images of mouse hearts; **(B)** Quantitative analysis of echocardiographic parameters: E/A ratio, EF, FS, LVIDs and LVIDd; **(C)** Representative images of HE staining of mouse myocardium; **(D)** Serum LDH levels; **(E)** Representative images of Masson’s trichrome staining and quantitative analysis of myocardial interstitial collagen deposition. Scale bars: 20 μm **P < 0.01 vs. CTL group; #*P* < 0.05, ##*P* < 0.01 vs. DCM, group, For echocardiographic parameters: n = 8; for serum LDH and collagen quantification: n = 4, Data are presented as mean ± SEM.

### 3.3 CA inhibited lipid deposition in the hearts of mice with DCM by lipophagy

To verify LD accumulation, cardiac TG levels were measured and Oil Red O staining was performed frozen myocardial sections from DCM mice. Compared with the CTL group, the DCM group showed significantly increased cardiac TG levels and Oil-Red O-positive area, both of which were significantly reduced by CA treatment (Figures 3A,B). Lipid deposition in myocardial tissue was further examined by TEM. TEM analysis revealed a decreased number of LDs within cardiomyocytes in the CA-treated group, in which LDs were observed to be encapsulated by autophagosomes and degraded (Figure 3C). The expression levels of autophagy-related proteins in mouse cardiac tissues were then measured. The DCM group exhibited a decreased LC3-II/LC3-I ratio and increased p62 protein levels, indicative of impaired autophagic flux, whereas CA treatment significantly restored autophagic flux (Figure 3D). Collectively, these results indicate that CA mitigates abnormal cardiac lipid deposition in DCM mice by inducing lipophagy.

**FIGURE 3:**
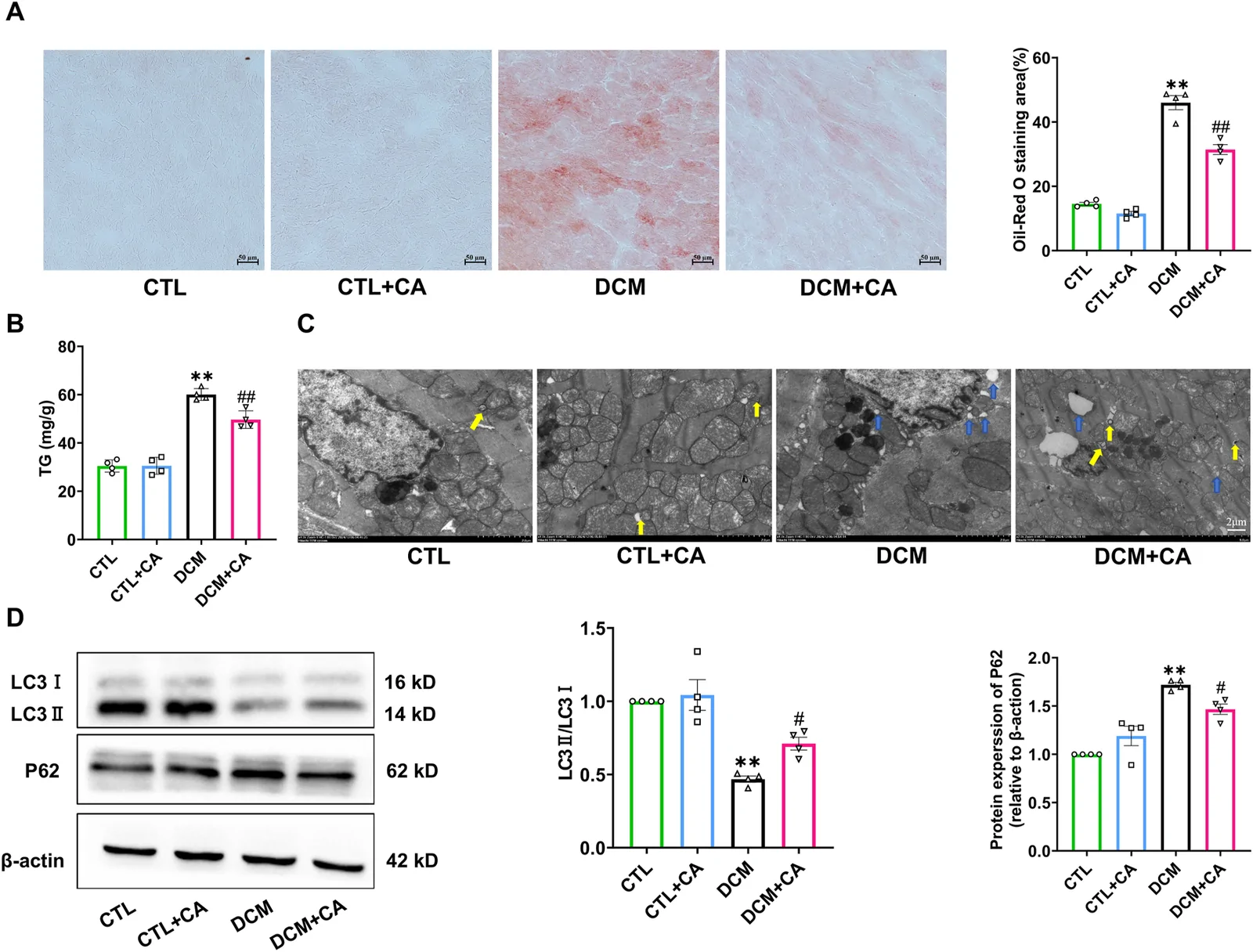
CA can reduce lipid content in the heart through lipid autophagy. **(A)** Oil-Red staining representative image. Scale bar, 50 μm **(B)** Quantitative analysis of TG content. **(C)** Transmission electron microscope representative image, The yellow arrow indicates the autophagosome and autolysosome, the blue arrow indicates the LD. Scale bar, 2 μm **(D)** Protein levels of LC3-II/LC3-I ratio and P62 by Western blot analysis. **P < 0.01 vs. CTL, #*P* < 0.05, ##*P* < 0.01 vs. DCM, n = 4, Data are presented as the mean ± SEM.

### 3.4 CA inhibited HGHL-induced lipid deposition in NMCMs by enhancing lipophagy

To further validate the regulatory effect of CA on lipid deposition observed in DCM mice, an *in vitro* DCM model was established by exposing isolated NMCMs to HGHL. The MTT assay showed that 0.25–5 μmol/L. CA·had no significant effect on cardiomyocyte viability versus the NG group, while 10 μmol/L CA markedly suppressed cell viability, confirming this low-concentration range as the safe dose of CA. Further, 5·μmol/L CA significantly reversed the·HGHL-induced reduction in cell viability. Thus, 5 μmol/L was chosen as the CA, intervention, concentration for subsequent experiments (Figures 4A,B). Consistent with the *in vivo* results, LDH release into the culture supernatant was significantly increased in HGHL-treated NMCMs, and this effect was markedly mitigated by CA treatment (Figure 4C). Intracellular lipid deposition was then examined. Bodipy staining revealed a significant increase in lipid fluorescence intensity in the HGHL group compared with the NG group, which was significantly reduced by CA treatment (Figure 4D). Co-labeling of HGHL-treated NMCMs with Bodipy and LC3 showed colocalization between LDs and LC3, and this colocalization was further enhanced by CA treatment (Figure 4E). Additionally, CA treatment significantly upregulated cellular autophagic flux in HGHL-injured NMCMs (Figure 4F).

**FIGURE 4:**
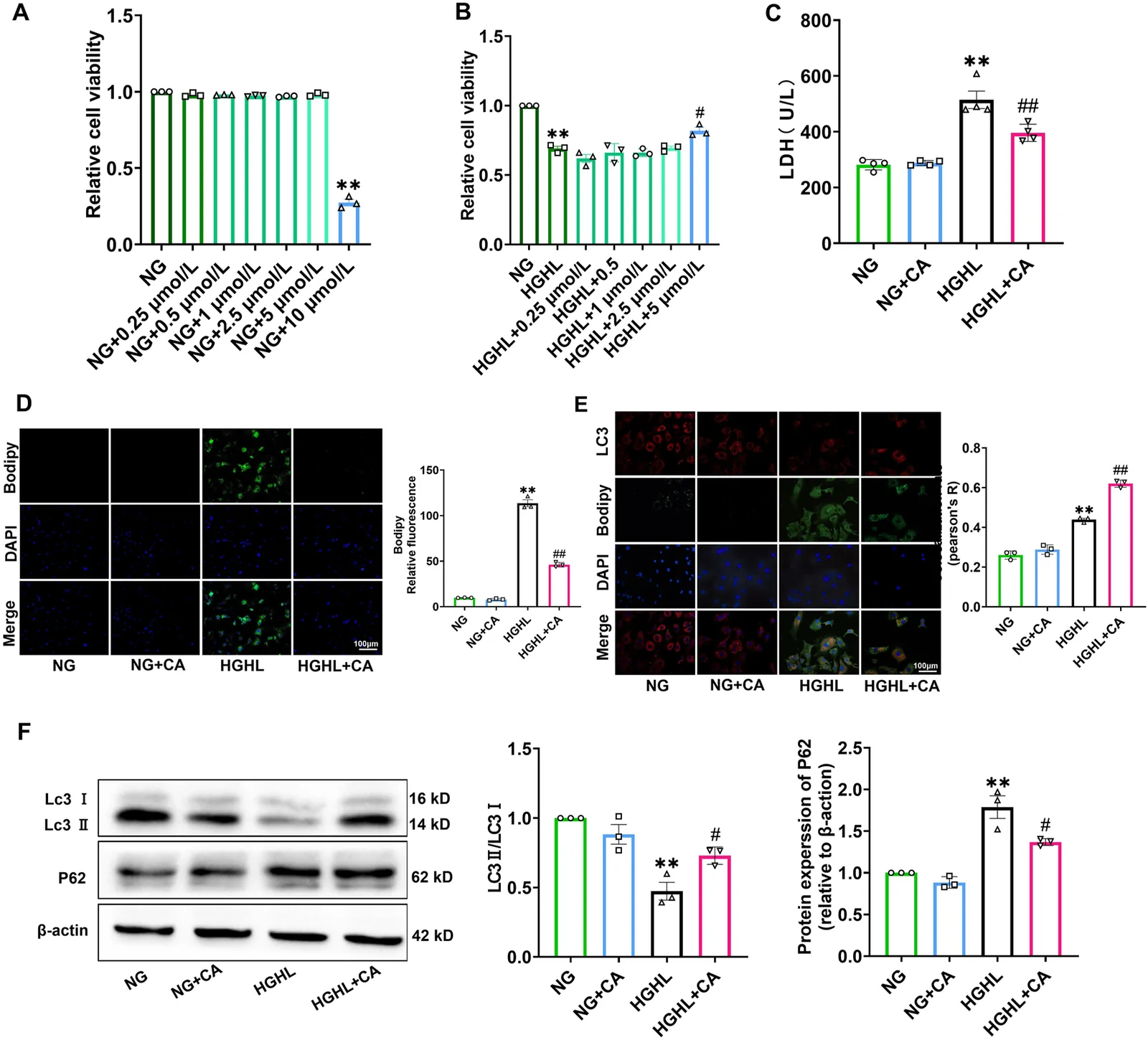
CA reduced lipid deposition in NMCMs by enhancing lipophagy. **(A,B)** Cell viability of NMCMs detected by MTT assay; **(C)** LDH release (marker of cardiomyocyte injury); **(D)** Representative images of Bodipy staining for intracellular lipid deposition; **(E)** Representative images of colocalization between LC3 and LDs; **(F)** LC3-II/LC3-I ratio and p62 protein levels detected by Western blotting. **P < 0.01 vs. NG, #*P* < 0.05, ##*P* < 0.01 vs. HGHL, n = 3, Data are presented as the mean ± SEM.

### 3.5 CA interacted with Rab7a

To elucidate the molecular mechanism by which CA regulated lipophagy in DCM, bioinformatics intersection analysis was performed to identify genes associated with both lipid deposition and DCM (Figure 5A). Among the 4,873 analyzed genes, 185 were the overlapping genes. KEGG and GO enrichment analysis revealed that these overlapping genes were most significantly enriched in the Lipid and Atherosclerosis pathway and lipid catabolic process (Figures 5B,C), while only three genes were associated with both pathways (Figure 5D). Among these three candidate genes, Rab7a was the only one functionally associated with autophagy. Molecular docking using AutoDock Vina predicted that CA may bind to Rab7a with high affinity in the region of Lys127, providing a plausible structural basis for the CA-mediated decrease in Rab7a ubiquitination, suggesting that CA may regulate Rab7a ubiquitination by occupying this functional site (Figure 5E). Compared with the CTL group, Rab7a protein levels were significantly reduced in both DCM mouse hearts and HGHL-treated NMCMs, and this reduction was completely rescued by CA treatment (Figures 5F,G). Co-IP assays showed that CA treatment significantly inhibited the HGHL-induced increase in Rab7a ubiquitination (Figure 5H).

**FIGURE 5:**
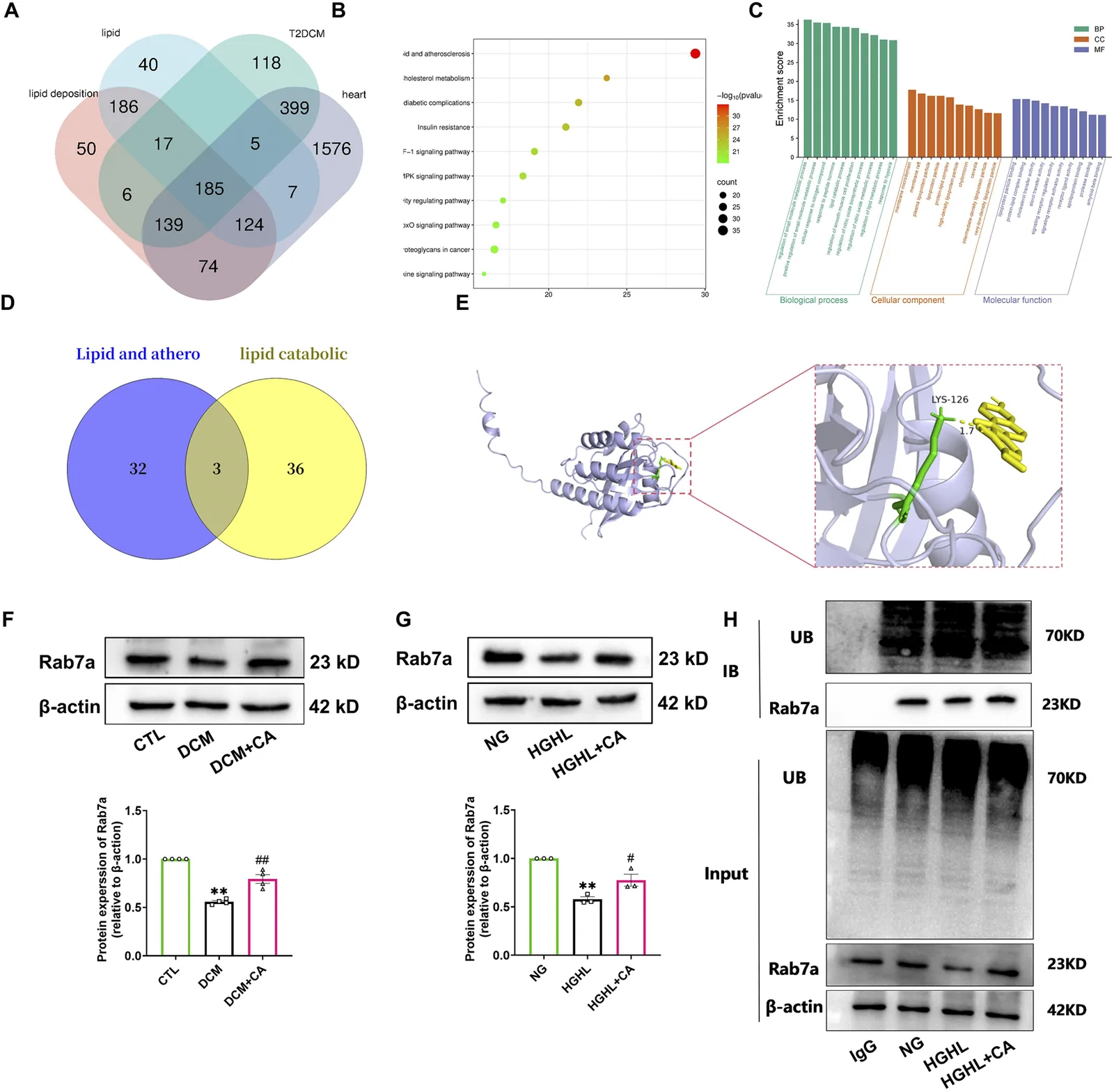
CA interacted with Rab7a and regulated its expression and ubiquitination. **(A)** Venn diagram showing the intersection of genes associated with lipid metabolism, diabetic cardiomyopathy, lipid deposition and cardiac proteins; **(B)** Bubble plot of KEGG pathway enrichment analysis; **(C)** Bar plot of GO biological process enrichment analysis; **(D)** Venn diagram showing the intersection of genes enriched in the Lipid and Atherosclerosis pathway and lipid catabolic process; **(E)** Molecular docking visualization of CA binding to Rab7a; **(F)** Rab7a protein levels in mouse cardiac tissues detected by Western blotting. **P < 0.01 vs. CTL, ##*P* < 0.01 vs. DCM, n = 4; **(G)** Rab7a protein levels in NMCMs detected by Western blotting. **P < 0.01 vs. NG, #*P* < 0.05 vs. HGHL, n = 3; Data are presented as the mean ± SEM. **(H)** Representative images of Rab7a ubiquitination detected by Co-IP assay.

### 3.6 Rab7a knockdown impaired lipophagy and exacerbates NMCMs injury

To verify that Rab7a is required for CA-mediated lipophagy, NMCMs were transfected with three different Rab7a-specific small interfering RNAs (si-Rab7a) and a negative control siRNA (NC). We selected si-Rab7a-2 (designated as SI-2), which exhibited the highest Rab7a knockdown efficiency of 75% (Figure 6A), was selected for subsequent experiments Functional evaluations revealed that Rab7a knockdown significantly exacerbated HGHL-induced NMCM injury, as indicated by increased LDH release in the HGHL + SI group compared with the HGHL + NC group (Figure 6B). Furthermore, Bodipy staining demonstrated that Rab7a silencing markedly aggravated HGHL-induced intracellular lipid accumulation (Figure 6C). Immunofluorescence co-staining showed that Rab7a depletion significantly reduced the colocalization between LC3 and LDs, indicating impaired lipophagy (Figure 6D). Consistently, Western blotting confirmed that Rab7a knockdown decreased the LC3-II/LC3-I ratio and increased p62 accumulation, further verifying inhibited autophagic flux (Figure 6E).

**FIGURE 6:**
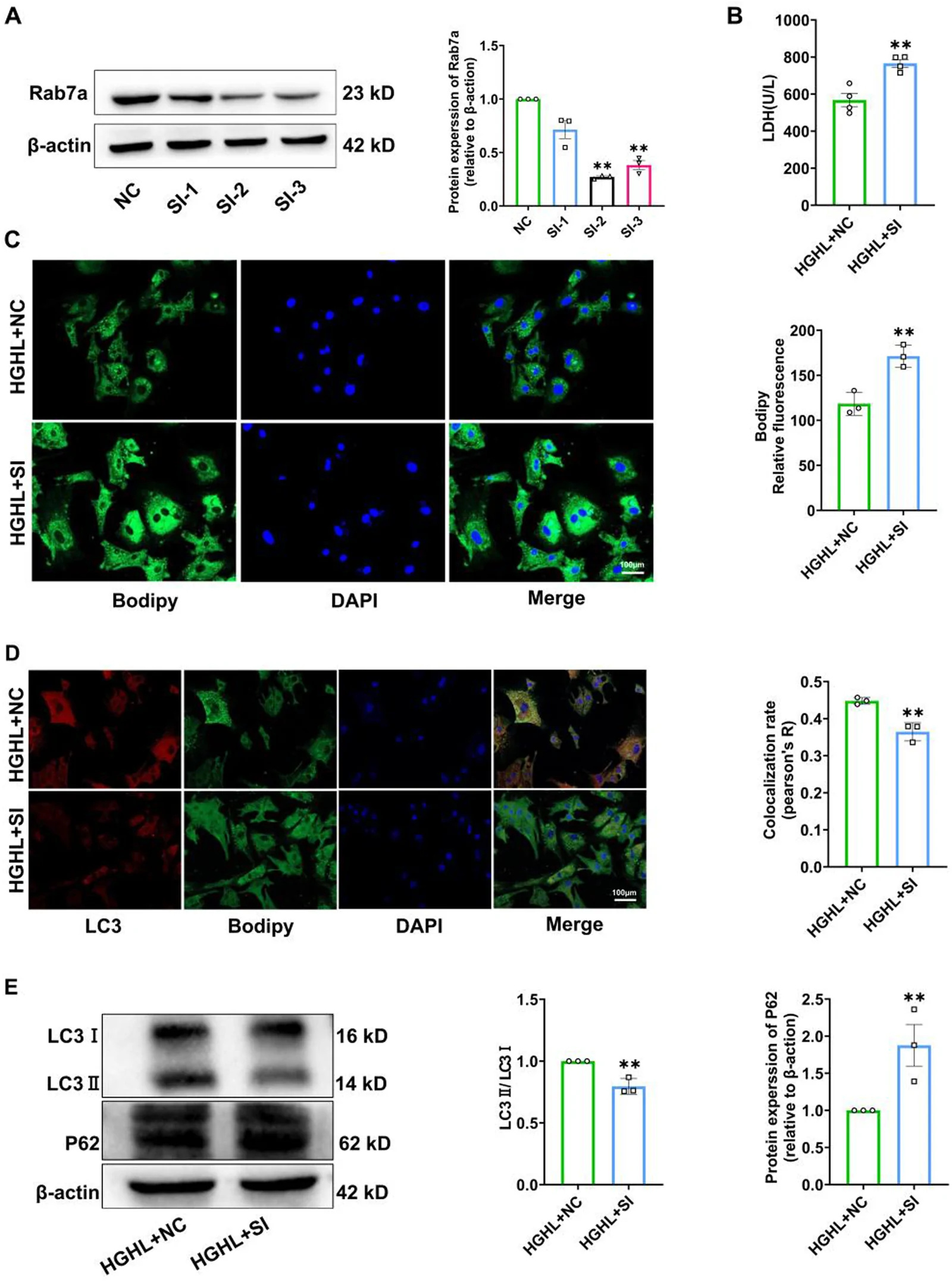
Rab7a knockdown impaired lipophagy and exacerbated HGHL-induced NMCM injury. **(A)** Rab7a protein expression levels after siRNA transfection; **P < 0.01 vs. HGHL + NC, n = 3; **(B)** LDH release (marker of cardiomyocyte injury); **P < 0.01 vs. HGHL + NC, n = 3; **(C)** Representative images of Bodipy staining for intracellular lipid deposition; **P < 0.01 vs. HGHL + NC, n = 3; **(D)** Representative images of colocalization between LC3 and LDs; **P < 0.01 vs. HGHL + NC, n = 3; **(E)** LC3-II/LC3-I ratio and p62 protein levels detected by Western blotting. **P < 0.01 vs. HGHL + NC, n = 3, Data are presented as the mean ± SEM.

### 3.7 Knockdown Rab7a abolished the cardioprotective effect of CA in NMCMs

To confirm that Rab7a mediates the cardioprotective effect of CA, NMCMs were transfected with Rab7a-specific siRNA, followed by HGHL stimulation and CA treatment for 24 h. The results showed that Rab7a knockdown abolished the protective effect of CA against HGHL-induced NMCM injury (Figure 7A). Furthermore, lipophagic capacity was significantly decreased in Rab7a-silenced cells, as evidenced by the restoration of intracellular lipid accumulation that was previously reduced by CA (Figures 7B–D).

**FIGURE 7:**
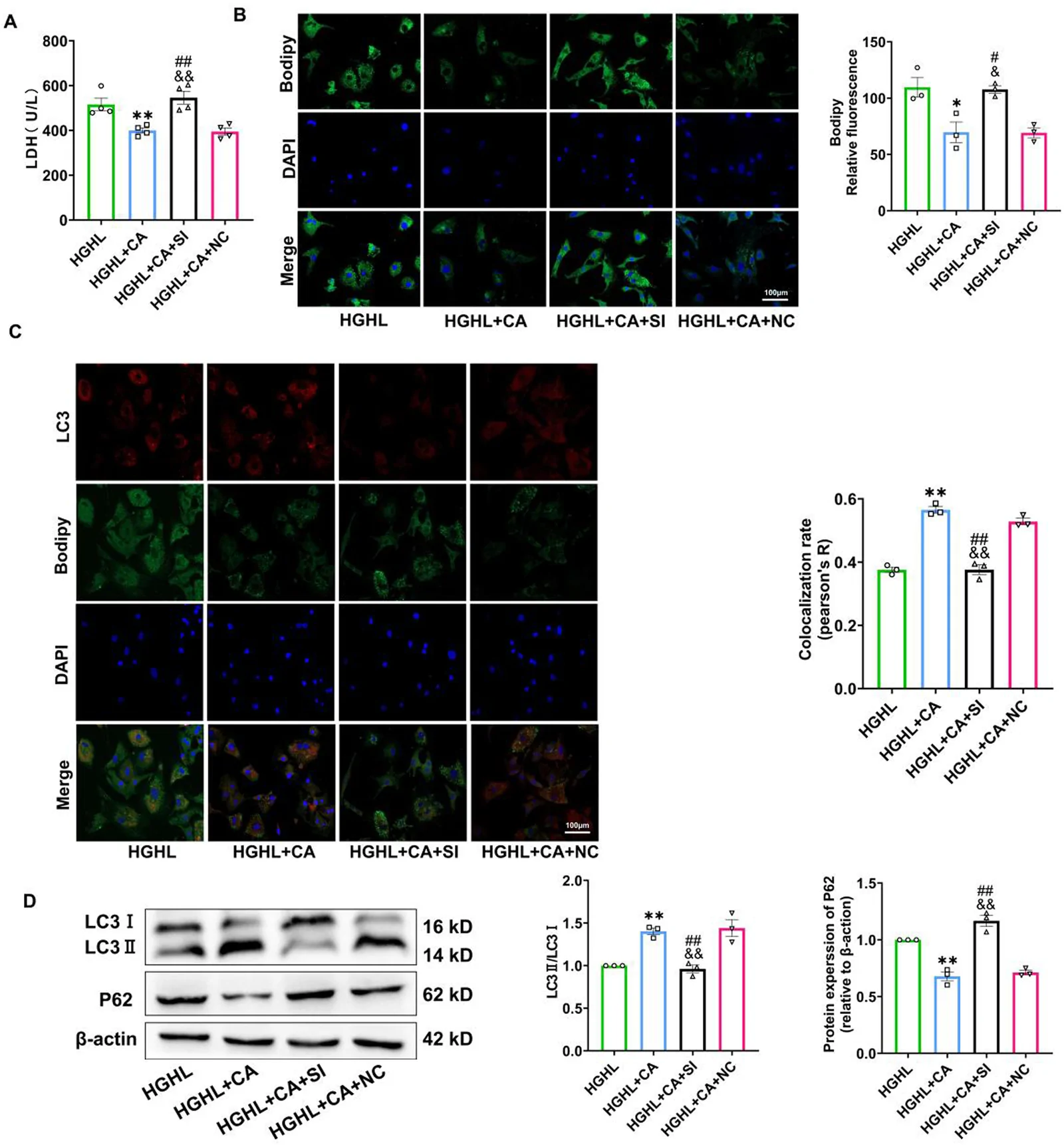
Rab7a knockdown abolished the cardioprotective and lipophagy-inducing effects of CA in NMCMs. **(A)** LDH release (marker of cardiomyocyte injury); **(B)** Representative images of Bodipy staining for intracellular lipid deposition; **(C)** Representative images of colocalization between LC3 and LDs; **(D)** LC3-II/LC3-I ratio and p62 protein levels detected by Western blotting. *P < 0.05, **P < 0.01 vs. HGHL, #*P* < 0.05, ##*P* < 0.01 vs. HGHL + CA + NC, &*p* < 0.05, &&*p* < 0.01 vs. HGHL + CA + NC, n = 3, Data are presented as the mean ± SEM.

**FIGURE 8:**
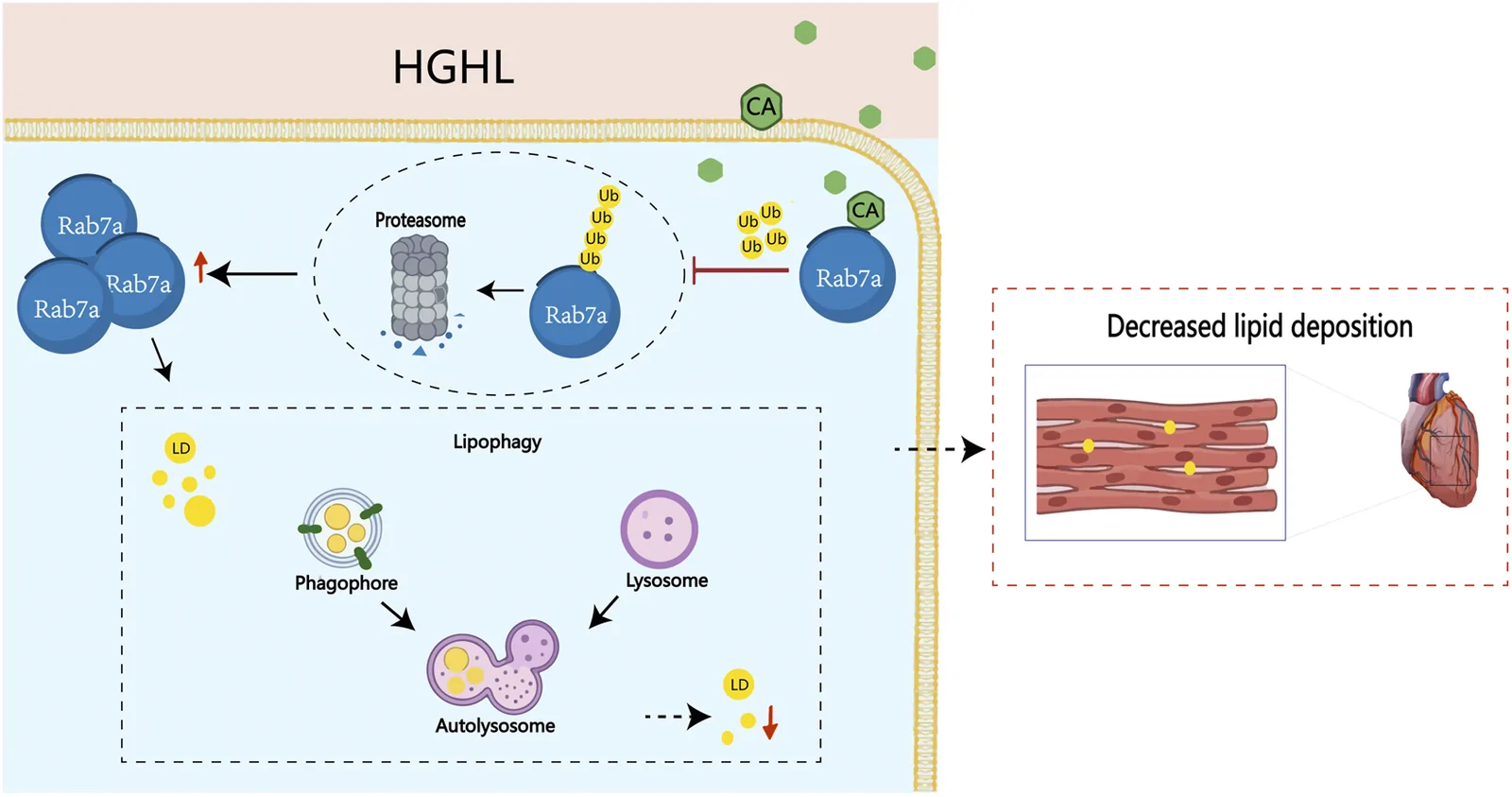
Protective mechanisms of CA in lipid deposition in diabetic mice.

Collectively, these results demonstrate that Rab7a is required for CA-mediated lipophagy induction and its subsequent cardioprotective effects against HGHL-induced injury in NMCMs.

## 4 Discussion

DCM is a leading cause of heart failure in diabetic patients, and its pathogenesis is closely associated with increased myocardial lipid deposition. Under lipid overload, TG accumulate abnormally in cardiomyocytes in the form of LDs (Plötz and Lenzen, 2024). Excessive accumulation of lipids and their metabolites within cardiomyocytes directly impairs cardiac function. In diabetes, TG hydrolysis releases free fatty acids, which induce oxidative stress, activate the NLRP3 inflammasome, and generate toxic lipid intermediates including diacylglycerol and ceramide. These events trigger endoplasmic reticulum stress, mitochondrial dysfunction, and apoptosis (Liu et al., 2025), ultimately leading to myocardial fibrosis, ventricular stiffening, and left ventricular dysfunction. Consistently, the present study confirmed that DCM mice exhibited overt cardiac dysfunction, manifested as decreased E/A ratio, EF, and FS, increased LVIDd/LVIDs, and elevated LDH release, along with myocardial disarray and excessive collagen deposition. Myocardial TG content and the positive area of Oil-Red O staining were also markedly increased. Similarly, HGHL treatment reduced viability and induced lipid accumulation and LDH release in cardiomyocytes.

Lipophagy, a selective autophagy subtype that degrades intracellular LDs, is pivotal for maintaining lipid homeostasis in cardiomyocytes (Wang and Chen, 2021). Dysregulation of LD autophagy leads to ectopic lipid accumulation in cardiomyocytes, triggering lipotoxicity and exacerbating cellular injury (Yang Y. et al., 2018). In this study, DCM model mice exhibited significant myocardial LD accumulation and suppressed autophagy levels, while cardiomyocytes treated under HGHL conditions similarly displayed markedly inhibited LD autophagy. Consequently, targeted activation of LD autophagy to eliminate excess lipids has emerged as a crucial strategy for DCM prevention and treatment.

CA, a predominant isoflavone extracted from *Astragalus membranaceus*, has garnered widespread recognition for its potent cardioprotective effects. Previous research has demonstrated that CA can mitigate myocardial fibrosis following myocardial infarction by restoring the homeostasis of fatty acid metabolism (Chen et al., 2022). Given that disordered lipid metabolism and excessive lipid deposition are recognized as key pathological drivers of DCM, it remains largely unclear whether CA exerts cardioprotective actions by ameliorating lipotoxicity and reducing myocardial lipid accumulation. The present study demonstrated that CA improved cardiac function, alleviated myocardial disarray and interstitial fibrosis, and decreased myocardial TG accumulation in DCM mice, with *in vitro* experiments consistently showing that CA also enhanced cell viability and reduced lipid deposition in HGHL-treated cardiomyocytes.

Modulating autophagy activity is an important way for CA to exert its cardiovascular pharmacological effects. Previous studies have shown that CA can reduce doxorubicin-induced cardiac injury by regulating cardiomyocyte autophagy (Lu et al., 2021; Zhang et al., 2022). In an atherosclerosis model, CA has also been confirmed to enhance autophagy activity by regulating the interaction between KLF2 and MLKL (Ma et al., 2022). Could CA, through its regulatory effects on autophagy, particularly lipophagy, confer protection against the pathological processes in DCM? Here, the present study further identified that CA activates lipophagy, a selective form of autophagy targeting LDs. CA treatment led to a notable reduction in myocardial LDs and a significant increase in autolysosome generation. Concurrently, CA treatment elevated the LC3-II/LC3-I ratio, decreased P62 protein levels, and enhanced the colocalization of LC3 with LDs. It is important to note that the conclusion of enhanced lipophagy in this study is supported by a convergent set of morphological, molecular, and functional evidence, rather than relying solely on global autophagic flux markers. Specifically, transmission electron microscopy directly visualized lipid droplets encapsulated within double-membrane autophagosomal structures in CA-treated myocardial tissue, which is regarded as the gold-standard morphological assay for confirming lipophagy. Furthermore, co-immunofluorescence staining showed increased recruitment of LC3 to the surface of lipid droplets, providing direct molecular evidence for the specific targeting of autophagic machinery to lipids. These findings are consistent with the functional lipid clearance endpoints, including reduced myocardial triglyceride content and Oil Red O staining. These findings collectively substantiate that CA effectively enhances lipophagic activity within cardiomyocytes.

The initiation and execution of LD autophagy rely on a precise molecular regulatory network. Identifying the key regulatory molecules targeted by CA in this network is essential for in-depth exploration of its pharmacological mechanisms. In this study, the autophagy-related small GTPase Rab7a was identified through bioinformatics analysis. As a member of the small GTPase family, Rab7a plays a dual role in autophagy. It not only participates in the biogenesis of autophagosomes, but also mediates the fusion between autophagosomes and lysosomes (Li et al., 2023). Molecular docking results indicated that CA suggested a potential interaction between CA and Rab7a in the region of Lys127, suggesting that CA might interfere with the ubiquitination process by occupying this site.

Ubiquitin-dependent degradation is a major pathway for protein turnover and a key post-translational mechanism regulating the stability of many autophagy-related proteins. In this study, Rab7a protein levels were significantly reduced in myocardial tissues of DCM mice and in HGHL-treated cardiomyocytes, and this reduction was reversed by CA treatment. Co-immunoprecipitation showed that Rab7a ubiquitination was markedly increased under HGHL conditions and was suppressed by CA. Functionally, Rab7a knockdown exacerbated HGHL-impaired lipophagy and abolished the CA-mediated enhancement of lipophagy. Together, these data support a model in which CA enhances lipophagy and reduces lipid accumulation by inhibiting ubiquitination-dependent degradation of Rab7a and thereby upregulating its protein expression.

Recent evidence has linked Rab7-family signaling to cardiac lipid catabolism. In a mouse model of DCM, activation of Rab7-Rilp-mediated microlipophagy alleviated lipotoxicity by promoting lysosomal clearance of LDs (Ke et al., 2024). Our findings complement this evidence from a different angle: rather than modulating the effector arm of Rab7a, we found that CA preserves Rab7a protein abundance by suppressing its ubiquitination-dependent degradation, thereby sustaining autophagosome maturation and autophagosome-lysosome fusion. In parallel, several natural compounds have been shown to activate lipophagy through distinct molecular nodes, including TFEB mobilization by nimbolide in atherosclerosis (Qin et al., 2026) and SIRT3-mediated lipophagy remodeling by berberine in DCM (Chen et al., 2025).

Despite these insights, several limitations should be noted. Neonatal cardiomyocytes differ metabolically from adult cardiomyocytes, which limits direct extrapolation of our *in vitro* mechanistic findings. In adult DCM mice, the *in vivo* assessment was limited to cardiac function, lipid deposition, and Rab7a protein levels; Rab7a downregulation and its restoration by CA were confirmed. The detailed mechanistic experiments, including Rab7a ubiquitination, lipophagic flux, and Rab7a knockdown, were performed in neonatal cardiomyocytes. Future validation in adult primary cardiomyocytes or cardiomyocyte-specific models is needed.

To the best of the authors’ knowledge, the present study demonstrates for the first time that CA alleviates myocardial injury in diabetic mice and HGHL-challenged cardiomyocytes by enhancing lipophagy and reducing intracellular lipid accumulation. The present study further clarifies that CA may exert these effects by suppressing Rab7a ubiquitination and proteasomal degradation, thereby stabilizing Rab7a protein expression and promoting lipophagic flux. However, whether CA prolongs Rab7a half-life and inhibits K48-linked ubiquitination, and which upstream regulators, post-translational modifications, E3 ligases, or deubiquitinating enzymes are involved, remain to be determined. Likewise, rescue experiments using siRNA-resistant Rab7a or Rab7a overexpression are needed to confirm causality. In addition, whether CA also regulates fatty acid uptake or oxidation remains unknown and warrants further investigation. Collectively, these findings not only uncover a novel pharmacological mechanism of CA in ameliorating DCM, but also establish a theoretical foundation for the prevention and treatment of DCM centered on the modulation of lipophagy.

## 5 Conclusion

The present study demonstrates that CA exerts significant cardioprotective effects against diabetic cardiomyopathy (DCM) both *in vivo* and *in vitro*. Mechanistically, CA may interact with Rab7a, potentially in the region of Lys127, inhibits its ubiquitin-mediated degradation, and thereby upregulates Rab7a protein expression. This leads to enhanced autophagosome-lysosome fusion, restored lipophagic flux, reduced intracellular lipid accumulation, and ultimately attenuated myocardial lipotoxicity and injury.

CA inhibits Rab7a ubiquitination and upregulates Rab7a protein expression, suggesting that CA may stabilize Rab7a by suppressing ubiquitin-proteasome-mediated degradation.

These findings reveal a novel mechanism by which CA regulates cardiomyocyte lipid metabolism, and identify Rab7a-mediated lipophagy as a critical therapeutic target for DCM. A schematic summary of the proposed protective mechanism of CA in diabetic cardiomyopathy, involving Rab7a regulation, enhanced lipophagy, and reduced myocardial lipid accumulation, is presented in Figure 8. Given that DCM remains a major cause of mortality in diabetic patients with no specific effective therapies currently available, CA shows promising potential as a natural cardioprotective agent for the prevention and treatment of DCM.

## Data Availability

The raw data supporting the conclusions of this article will be made available by the authors, without undue reservation.

## References

[B1] Berardi D. Bock-Hughes A. Terry A. Drake E. Bozek G. Macleod F. (2022). Lipid droplet turnover at the lysosome inhibits growth of hepatocellular carcinoma in a BNIP3-dependent manner. Sci. Adv. 8 (41), eabo2510. 10.1126/sciadv.abo2510 36223464 PMC9555787

[B2] Chen G. Xu H. Xu T. Ding W. Zhang G. Hua Y. (2022). Calycosin reduces myocardial fibrosis and improves cardiac function in post-myocardial infarction mice by suppressing TGFBR1 signaling pathways. Phytomedicine 104, 154277. 10.1016/j.phymed.2022.154277 35752078

[B3] Chen W. Jin T. Xie Y. Zhong C. Gao H. Zhang L. (2025). Berberine partially ameliorates cardiolipotoxicity in diabetic cardiomyopathy by modulating SIRT3-mediated lipophagy to remodel lipid droplets homeostasis. Br. J. Pharmacol. 182 (20), 5038–5056. 10.1111/bph.70035 40222752

[B4] Christian S. (2009). Myocardial lipid accumulation and lipotoxicity in heart failure. J. Lipid Res. 50 (11), 2138. 10.1194/jlr.R001115 PMC2759818 19687505

[B5] Guan X. Liu Y. Xin W. Qin S. Gong S. Xiao T. (2022). Activation of EP4 alleviates AKI-to-CKD transition through inducing CPT2-mediated lipophagy in renal macrophages. Front. Pharmacol. 13, 1030800. 10.3389/fphar.2022.1030800 36467025 PMC9709464

[B6] HuQ. TanR. ZouY. LiC. FanM. KantawongF. (2023). Synergism of calycosin and bone marrow-derived mesenchymal stem cells to combat podocyte apoptosis to alleviate adriamycin-induced focal segmental glomerulosclerosis. World J. Stem Cells 15 (6), 617–631. 10.4252/wjsc.v18.i2.116824 37424951 PMC10324505

[B7] Kanamori H. Takemura G. Goto K. Tsujimoto A. Mikami A. Ogino A. (2015). Autophagic adaptations in diabetic cardiomyopathy differ between type 1 and type 2 diabetes. Autophagy 11 (7), 1146–1160. 10.1080/15548627.2015.1051295 26042865 PMC4590644

[B8] Ke J. Pan J. Lin H. Huang S. Zhang J. Wang C. (2024). Targeting Rab7-Rilp mediated microlipophagy alleviates lipid toxicity in diabetic cardiomyopathy. Adv. Sci. (Weinh) 11 (29), e2401676. 10.1002/advs.202401676 38837607 PMC11304244

[B9] Li Z. Lai M. Li J. Yang D. Zhao M. Wang D. (2023). RAB7A GTPase is involved in mitophagosome formation and autophagosome-lysosome fusion in N2a cells treated with the prion protein fragment 106-126. Mol. Neurobiol. 60 (3), 1391–1407. 10.1007/s12035-022-03118-5 36449254

[B10] Li Y. Liu S. Min X. Cheng H. Zhang X. Liu J. (2026). HSPA12A impairs lipophagy to exaggerate septic cardiomyopathy by promoting MTOR’s competition against LC3-II in binding with PNPLA2. Autophagy 22 (2), 371–390. 10.1080/15548627.2025.2600895 41358560 PMC12834148

[B11] Liu H. Bai X. Wei W. Li Z. Zhang Z. Tan W. (2022). Calycosin ameliorates bleomycin-induced pulmonary fibrosis via suppressing oxidative stress, apoptosis, and enhancing autophagy. Evid. Based Complement. Altern. Med. 2022, 9969729. 10.1155/2022/9969729 36267093 PMC9578840

[B12] Liu J. Fan X. Song Y. Zhao J. (2025). Triglyceride-based lipotoxicity in the pathophysiology of chronic diseases. Trends Endocrinol. Metab. S1043-2760 (25), 371–385. 10.1016/j.tem.2025.07.004 40796398

[B13] Lopaschuk G. D. Ussher J. R. Folmes C. D. L. Jaswal J. S. Stanley W. C. (2010). Myocardial fatty acid metabolism in health and disease. Physiol. Rev. 90 (1), 207–258. 10.1152/physrev.00015.2009 20086077

[B14] Lu X. Lu L. Gao L. Wang Y. Wang W. (2021). Calycosin attenuates doxorubicin-induced cardiotoxicity via autophagy regulation in zebrafish models. Biomed. Pharmacother. 137, 111375. 10.1016/j.biopha.2021.111375 33761601

[B15] Ma C. Wu H. Yang G. Xiang J. Feng K. Zhang J. (2022). Calycosin ameliorates atherosclerosis by enhancing autophagy via regulating the interaction between KLF2 and MLKL in apolipoprotein E gene-deleted mice. Br. J. Pharmacol. 179 (2), 252–269. 10.1111/bph.15720 34713437

[B16] MichalJ. KacperT. JolantaS. TarekM. KulbackaJ. (2024). Lipids associated with autophagy: mechanisms and therapeutic targets. Cell Death Discov. 10 (1), 460. 10.1038/s41420-024-02224-8 39477959 PMC11525783

[B17] Plötz T. Lenzen S. (2024). Mechanisms of lipotoxicity-induced dysfunction and death of human pancreatic beta cells under obesity and type 2 diabetes conditions. Obes. Rev. 25 (5), e13703. 10.1111/obr.13703 38327101

[B18] Qiang L. Li H. Wang Z. Wan L. Jiang G. (2022). Deconvoluting the complexity of autophagy in colorectal cancer: from crucial pathways to targeted therapies. Front. Oncol. 12, 1007509. 10.3389/fonc.2022.1007509 36172152 PMC9510924

[B19] Qin Y. Zhou Y. Liu C. Zou J. He C. (2026). Nimbolide competitively binds to the 14-3-3 epsilon with TFEB to promote lipophagy and ameliorate atherosclerosis. Phytomedicine 161, 158774. 10.1016/j.phymed.2026.158774 42705181

[B20] Singh R. Cuervo A. (2011). Autophagy in the cellular energetic balance. Cell Metab. 13 (5), 495–504. 10.1016/j.cmet.2011.04.004 21531332 PMC3099265

[B21] Tabibzadeh S. (2023). Role of autophagy in aging: the good, the bad, and the ugly. Aging Cell 22 (1), e13753. 10.1111/acel.13753 36539927 PMC9835585

[B22] Tang X. Yan X. Zhou H. Huang G. Niu X. Jiang H. (2022). Associations of insulin resistance and beta-cell function with abnormal lipid profile in newly diagnosed diabetes. CMJ 135 (21), 2554–2562. 10.1097/CM9.0000000000002075 35245924 10.1097/CM9.0000000000002075 PMC9944004

[B23] WangY. ChenC. (2021). Energy metabolism homeostasis in cardiovascular diseases. J. Geriatr. Cardiol. 18 (12), 1044–1057. 10.11909/j.issn.1671-5411.2021.12.006 35136399 10.11909/j.issn.1671-5411.2021.12.006PMC8782763

[B24] Wen T. Zheng L. Li X. Cheng D. Liu Y. Lu K. (2019). Endurance exercise resistance to lipotoxic cardiomyopathy is associated with cardiac NAD+/dSIR2/PGC-1α pathway activation in old drosophila. Biol. Open 8 (10), bio044719. 10.1242/bio.044719 31624074 PMC6826281

[B25] Wu G. Qi G. Liu Y. Gan J. Xie C. Wu Q. (2024). ER-α36 is involved in calycosin inhibition of IL-6 production in macrophages. J. Cell Mol. Med. 28 (1), e18037. 10.1111/jcmm.18037 37974543 PMC10805506

[B26] Xu X. Kobayashi S. Chen K. Timm D. Volden P. Huang Y. (2013). Diminished autophagy limits cardiac injury in mouse models of type 1 diabetes. J. Biol. Chem. 288 (25), 18077–18092. 10.1074/jbc.M113.474650 23658055 PMC3689952

[B27] Yang M. Zhang Y. Ren J. (2018). Autophagic regulation of lipid homeostasis in cardiometabolic syndrome. Front. Cardiovasc Med. 5, 38. 10.3389/fcvm.2018.00038 29774216 PMC5943591

[B28] Yang Y. Sun T. Li X. Chen Y. Wang X. (2018). Protective effect of angiotensin-(1-7) against hyperglycaemia-induced injury in H9c2 cardiomyoblast cells via the PI3K/Akt signaling pathway. Int. J. Mol. Med. 41 (3), 1283–1292. 10.3892/ijmm.2017.3322 29286068 PMC5819934

[B29] Yosri H. El-Kashef D. H. El-Sherbiny M. Said E. Salem H. A. (2022). Calycosin modulates NLRP3 and TXNIP-mediated pyroptotic signaling and attenuates diabetic nephropathy progression in diabetic rats; an insight. Biomed. Pharmacother. 155, 113758. 10.1016/j.biopha.2022.113758 36271546

[B30] Zhang Y. Bao M. Dai M. Wang X. He W. Tan T. (2015). Cardiospecific CD36 suppression by lentivirus-mediated RNA interference prevents cardiac hypertrophy and systolic dysfunction in high-fat-diet induced obese mice. Cardiovasc Diabetol. 14, 69. 10.1186/s12933-015-0234-z 26036798 PMC4464858

[B31] Zhang L. Fan C. Jiao C. Zhang Q. Jiang H. Cui J. (2022). Calycosin alleviates doxorubicin-induced cardiotoxicity and pyroptosis by inhibiting NLRP3 inflammasome activation. Oxid. Med. Cell Longev. 2022, 1733834. 10.1155/2022/1733834 35035656 PMC8754606

