# Supplementary material for: Calycosin alleviates myocardial injury in diabetic mice by enhancing lipophagy in cardiomyocytes via Rab7a regulation
Source: Front Pharmacol. 2026 Oct 22;17:1880993. doi: 10.3389/fphar.2026.1880993 (PMC13735551; doi:10.3389/fphar.2026.1880993)
Supplement: Supplementary file 1 [file DataSheet1.pdf]

Figure 3-D

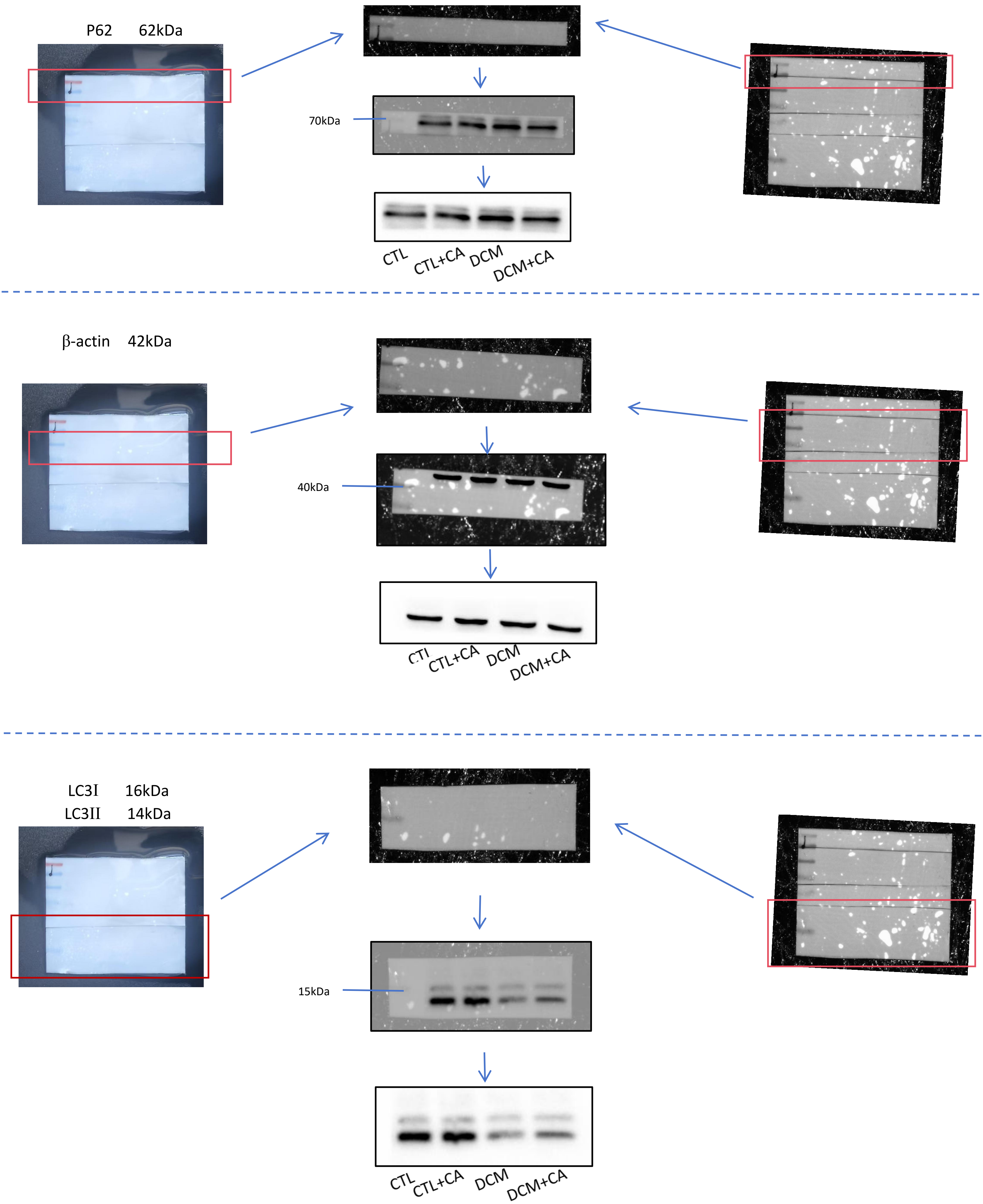

P62 62kDa
70kDa
CTL
CTL+CA
DCM
DCM+CA
β-actin 42kDa
40kDa
CTL
CTL+CA
DCM
DCM+CA
LC3I 16kDa
LC3II 14kDa
15kDa
CTL
CTL+CA
DCM
DCM+CA

Figure 4-F

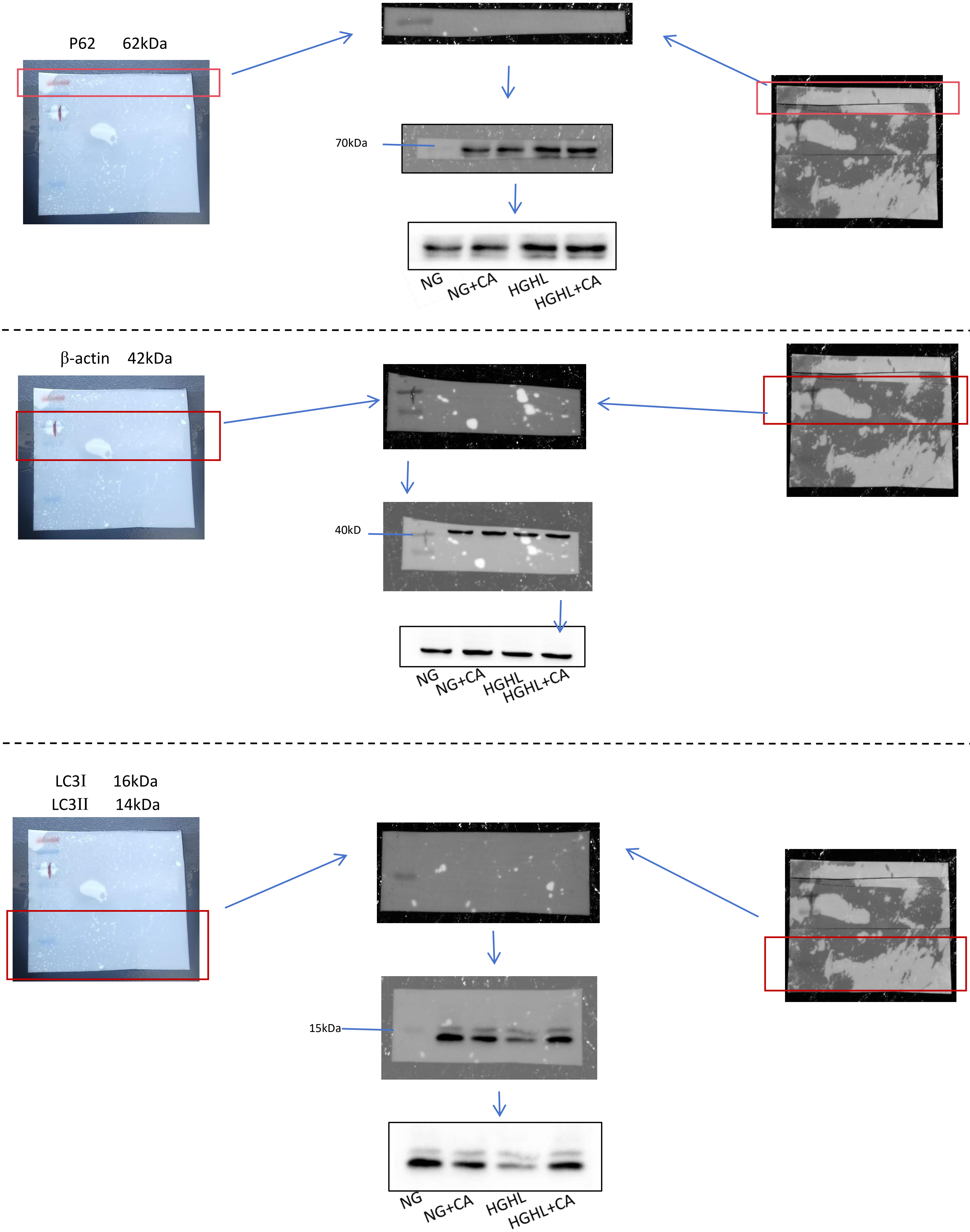

P62 62kDa
70kDa
NG
NG+CA
HGHL
HGHL+CA
β-actin 42kDa
40kD
NG
NG+CA
HGHL
HGHL+CA
LC3I 16kDa
LC3II 14kDa
15kDa
NG
NG+CA
HGHL
HGHL+CA

Figure 5-F

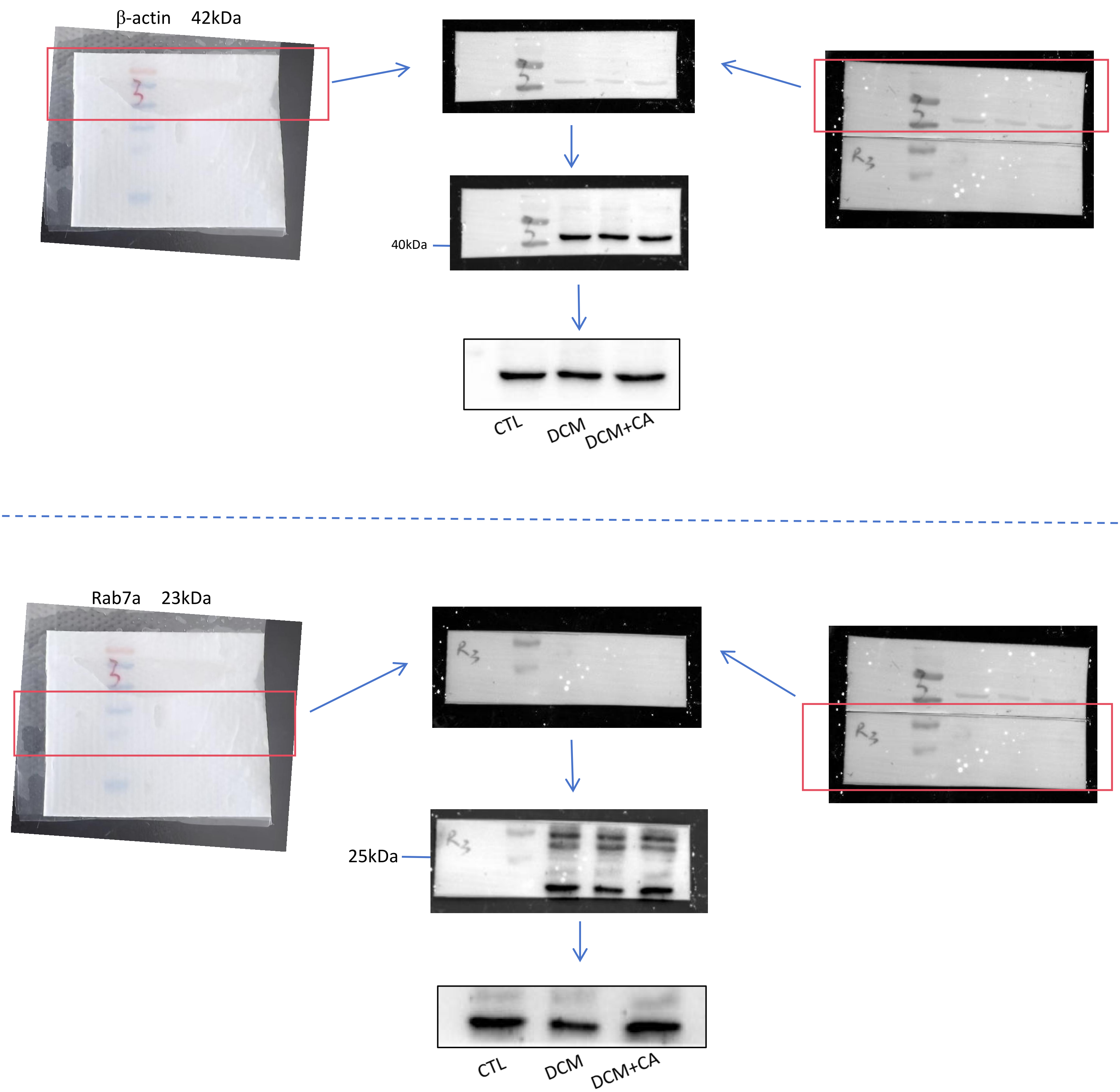
β-actin 42kDa
40kDa
CTL
DCM
DCM+CA
Rab7a 23kDa
25kDa
CTL
DCM
DCM+CA

Figure 5-G

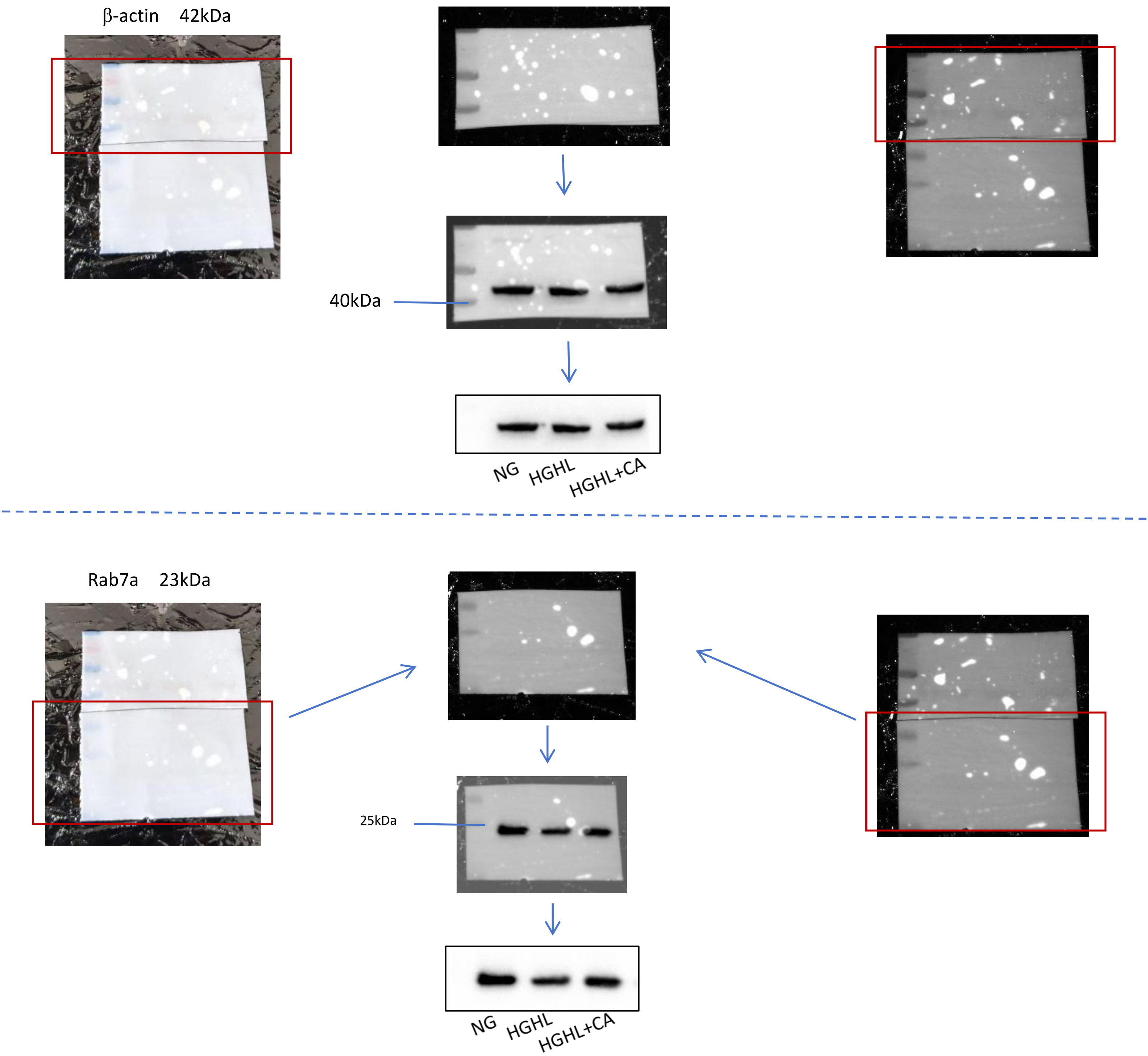
β-actin 42kDa
40kDa
NG
HGHL
HGHL+CA
Rab7a 23kDa
25kDa
NG
HGHL
HGHL+CA

Figure 5-H

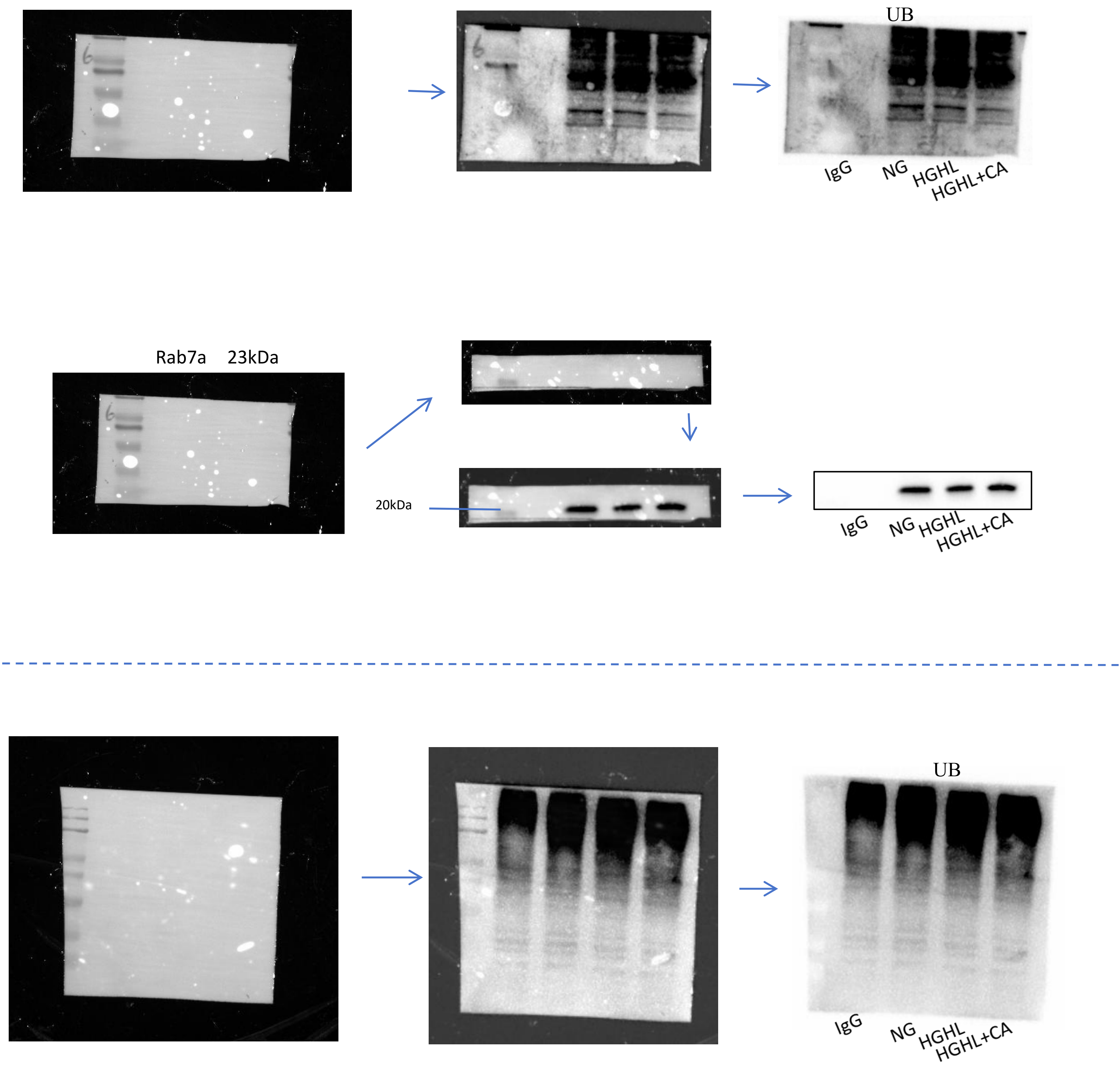
UB
IgG
NG
HGHL
HGHL+CA
Rab7a 23kDa
20kDa
IgG
NG
HGHL
HGHL+CA
UB
IgG
NG
HGHL
HGHL+CA

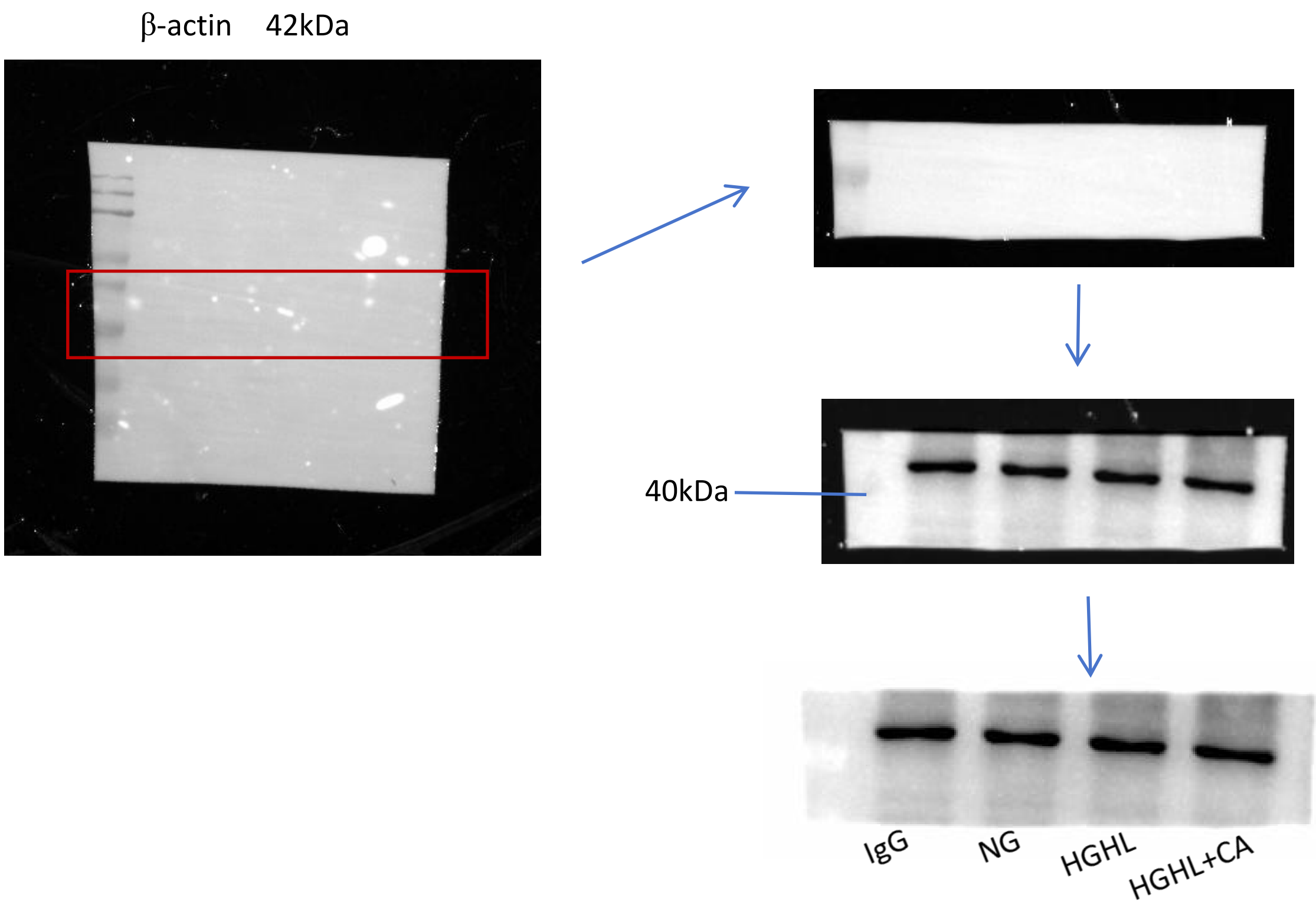
β-actin 42kDa
40kDa
IgG
NG
HGHL
HGHL+CA

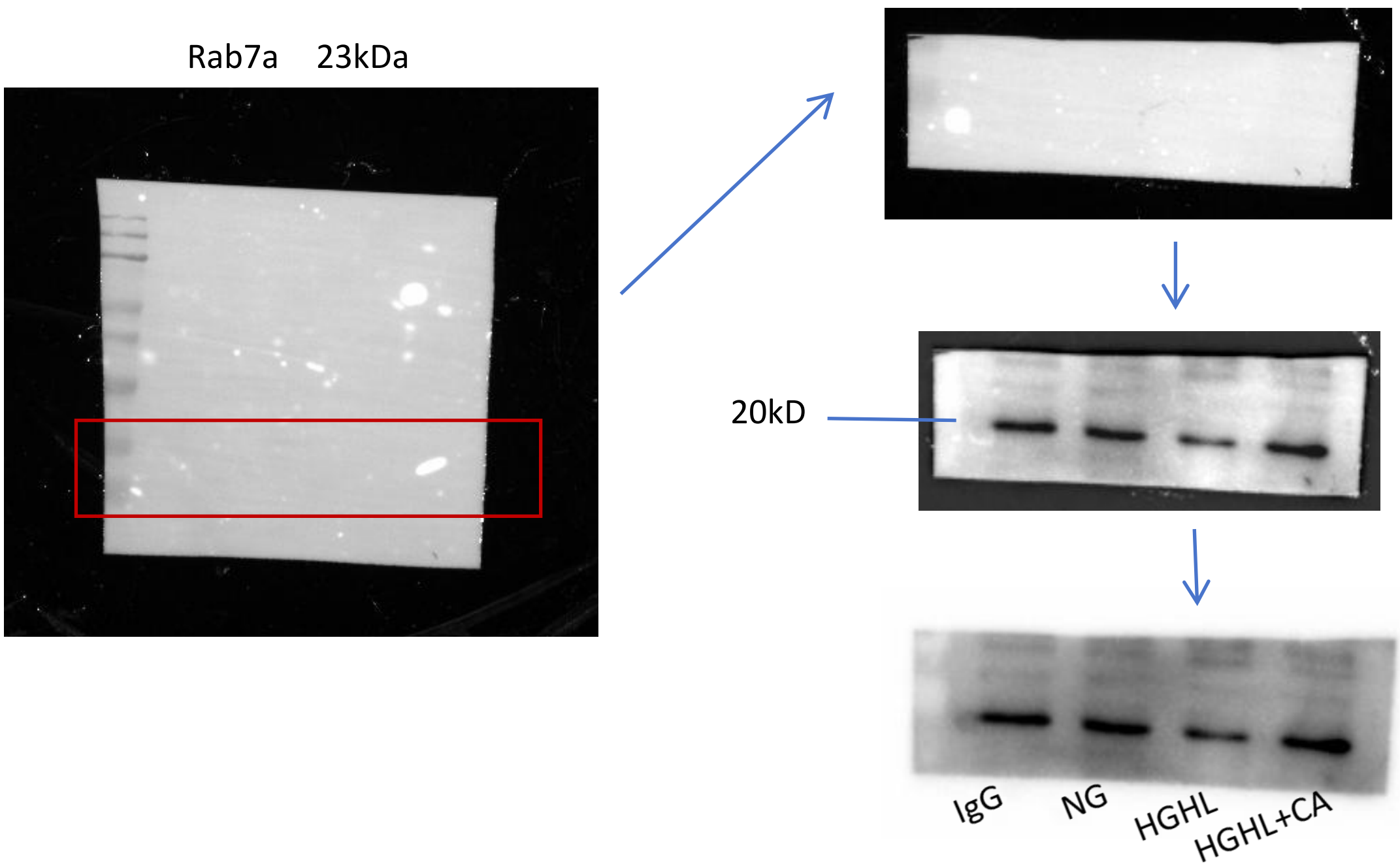
Rab7a 23kDa
20kD
IgG
NG
HGHL
HGHL+CA

Figure 6-A

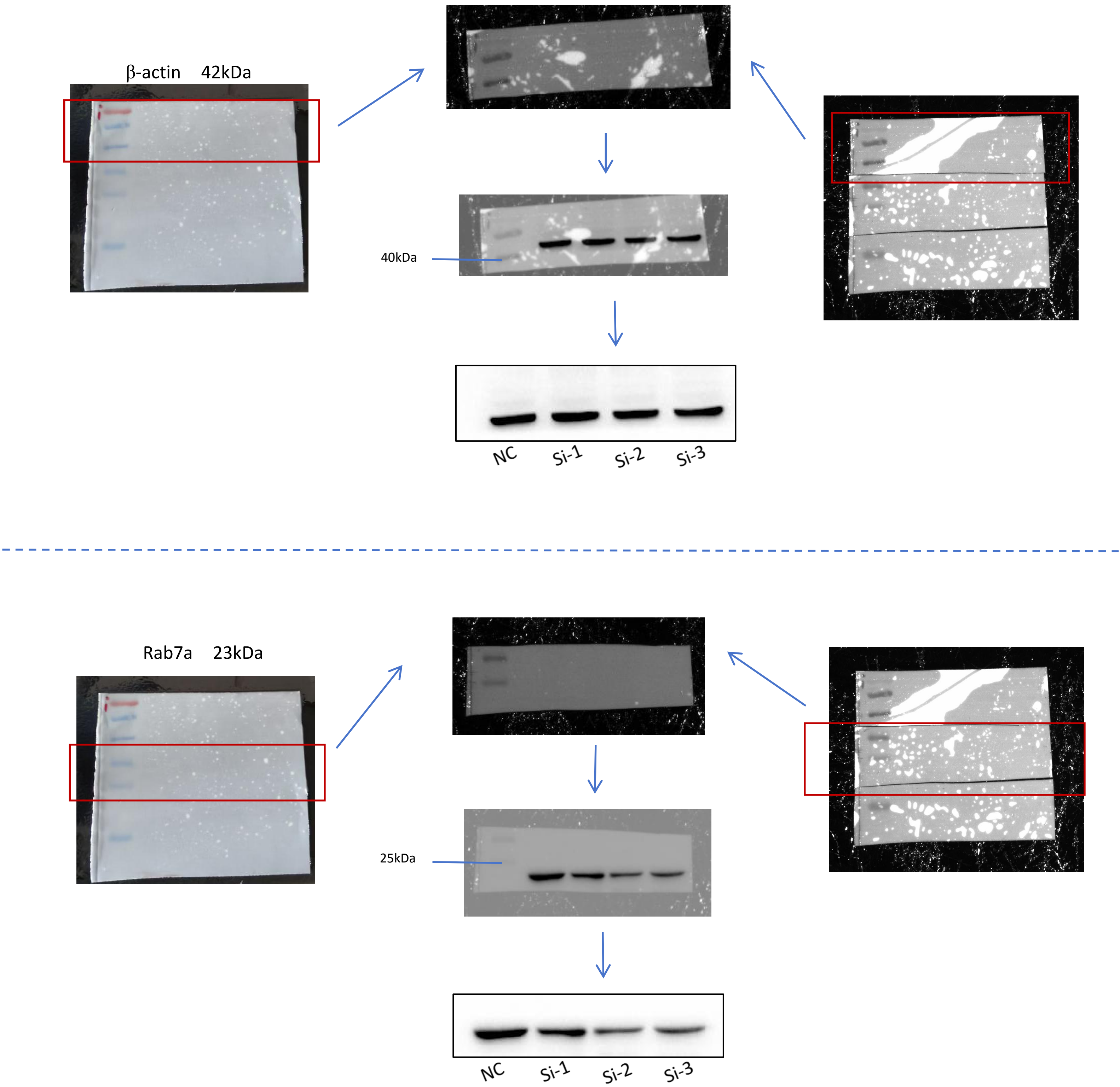
β-actin 42kDa
40kDa
NC
Si-1
Si-2
Si-3
Rab7a 23kDa
25kDa
NC
Si-1
Si-2
Si-3

Figure 6-E

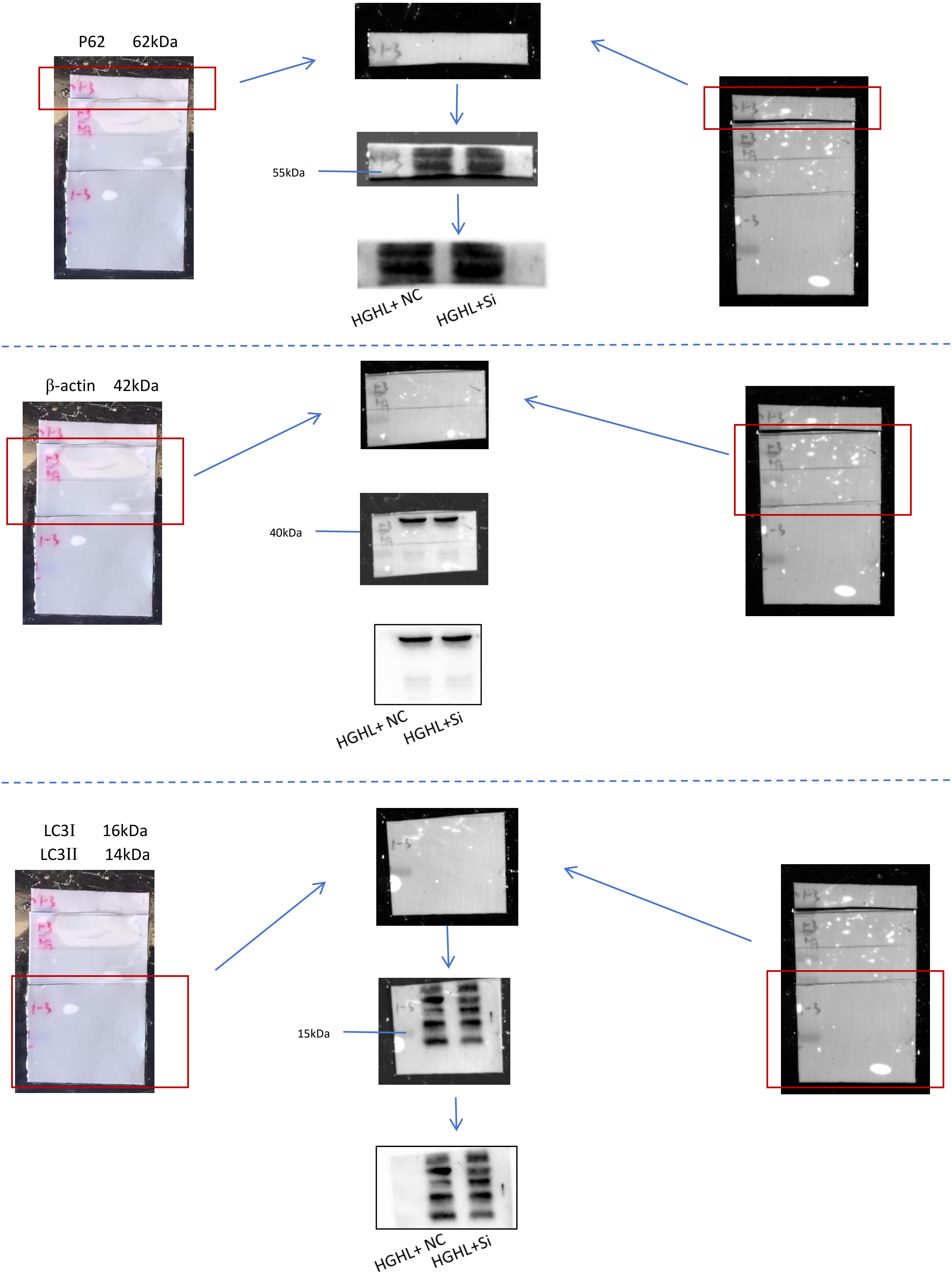
P62 62kDa
55kDa
HGHL+ NC
HGHL+Si
β-actin 42kDa
40kDa
HGHL+ NC
HGHL+Si
LC3I 16kDa
LC3II 14kDa
15kDa
HGHL+ NC
HGHL+Si

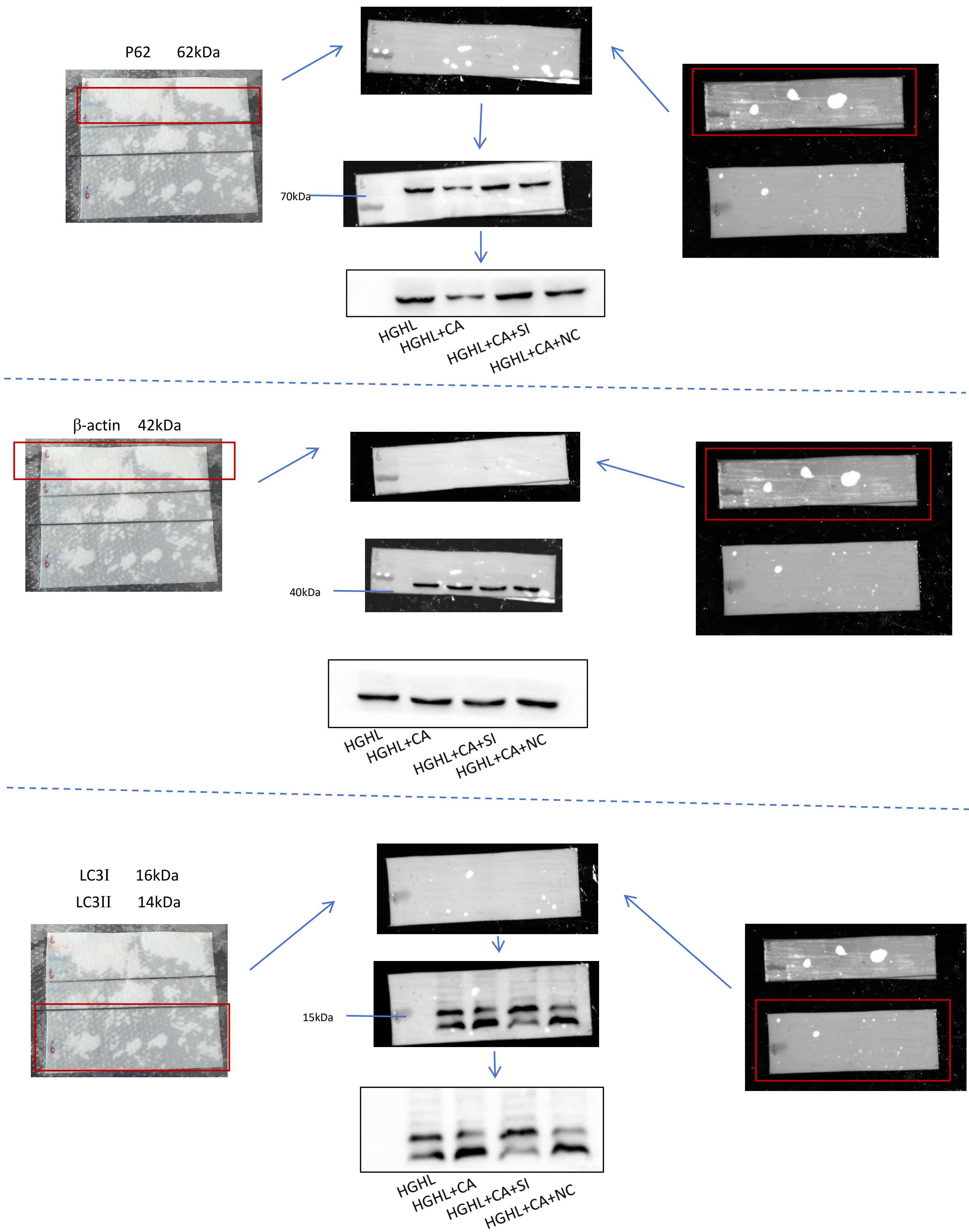
P62 62kDa
70kDa
HGHL
HGHL+CA
HGHL+CA+SI
HGHL+CA+NC
β-actin 42kDa
40kDa
HGHL
HGHL+CA
HGHL+CA+SI
HGHL+CA+NC
LC3I 16kDa
LC3II 14kDa
15kDa
HGHL
HGHL+CA
HGHL+CA+SI
HGHL+CA+NC

Frigue 7-D
